# The Gut Microbiota–Polyphenol–NLRP3 Inflammasome Axis: A Key Regulatory Network Linking Diet to Chronic Inflammation

**DOI:** 10.3390/nu18101483

**Published:** 2026-05-07

**Authors:** Laura Mosca, Cristina Pagano, Maria Giovanna Tafuri, Girolamo Di Maio, Claudia M. Rejano-Gordillo, Roberta Della Marca, Stefania D’Angelo, Marcellino Monda, Giovanni Messina, Rita Polito, Pasquale Perrone

**Affiliations:** 1 Department of Human Sciences and Promotion of the Quality of Life, San Raffaele University, 00166 Rome, Italy; laura.mosca@uniroma5.it; 2 Department of Molecular Medicine and Medical Biotechnology, University of Naples Federico II, 80131 Naples, Italy; cristina.pagano@unina.it; 3 Department of Literary, Pegaso University, 80143 Naples, Italy; mariagiovanna.tafuri@unipegaso.it; 4 Department of Psychology and Health Sciences, Pegaso Telematic University, 80143 Naples, Italy; girolamo.dimaio@unipegaso.it (G.D.M.); rita.polito@unipegaso.it (R.P.); 5 Department of Biochemistry, Molecular Biology and Genetics, University of Extremadura, 06006 Badajoz, Spain; claudiamrg@unex.es; 6 Department of Experimental Medicine, University of Campania “Luigi Vanvitelli”, 81100 Naples, Italy; roberta.dellamarca@unicampania.it; 7 Department of Medical, Movement, and Wellbeing Sciences, Parthenope University of Naples, 80133 Naples, Italy; stefania.dangelo@uniparthenope.it; 8 Unit of Dietetics and Sports Medicine, Section of Human Physiology, Department of Experimental Medicine, University of Campania “Luigi Vanvitelli”, 80138 Naples, Italy; marcellino.monda@unicampania.it (M.M.); giovanni.messina@unicampania.it (G.M.)

**Keywords:** chronic inflammation, dietary polyphenols, gut microbiota, intestinal barrier, microbial metabolites, NLRP3 inflammasome

## Abstract

**Background/Objectives**: Chronic low-grade inflammation, underpinned by persistent activation of the NLRP3 inflammasome, is a central pathological mechanism in non-communicable diseases including cardiovascular disease, type 2 diabetes, inflammatory bowel disease, and neurodegeneration. Dietary polyphenols have been consistently associated with reduced inflammatory burden; however, the mechanisms underlying these effects remain incompletely understood. This review aims to characterize the gut microbiota–polyphenol–NLRP3 inflammasome axis as a central regulatory network through which diet modulates innate immune signaling and chronic inflammatory tone. **Methods**: A comprehensive narrative review of the available literature was conducted, integrating evidence from mechanistic studies in cell culture and animal models, microbiome research, metabolomics, and human epidemiological and interventional data. **Results**: The gut microbiota emerges as a critical biochemical intermediary that transforms dietary polyphenols into bioactive metabolites, including urolithins, phenyl-γ-valerolactones, protocatechuic acid, and short-chain fatty acids, with enhanced bioavailability and potent inflammasome-modulating properties. These compounds suppress NLRP3 activation through multiple converging mechanisms, including inhibition of NF-κB-dependent priming, mitochondrial quality control via mitophagy, Nrf2-mediated antioxidant responses, and HDAC inhibition. Evidence across cardiovascular, metabolic, neurological, and respiratory disease models supports the translational relevance of this axis. **Conclusions**: The microbiota–polyphenol–NLRP3 axis functions as an integrated, self-regulated network in which each component simultaneously shapes and is shaped by the others: dysbiosis primes NLRP3 and depletes protective metabolites, while inflammasome hyperactivation further destabilises microbial ecology; polyphenol biotransformation by specific taxa interrupts this feed-forward loop at multiple nodes, restoring homeostasis.

## 1. Introduction

Chronic low-grade inflammation is recognized as a fundamental pathophysiological mechanism underlying the development and progression of a wide range of non-communicable diseases, including obesity, type 2 diabetes, cardiovascular disorders, inflammatory bowel disease (IBD), neurodegenerative diseases, and cancer [1]. Unlike acute inflammation, which is tightly regulated and resolves upon elimination of the triggering insult, chronic inflammation is characterized by persistent immune activation, metabolic stress, and tissue remodeling, ultimately leading to progressive organ dysfunction [2,3]. This inflammatory milieu is increasingly understood as the result of a continuous interplay between metabolic cues, immune signaling, and environmental factors, among which diet and the gut microbiota play a central role [4,5].

Within this complex network, innate immune sensors that detect both pathogen-associated molecular patterns (PAMPs) and danger-associated molecular patterns (DAMPs) are crucial for translating environmental and metabolic perturbations into inflammatory responses [6]. Among these sensors, the NOD-like receptor family pyrin domain-containing 3 (NLRP3) inflammasome has emerged as a master regulator of sterile and microbe-driven inflammation. NLRP3 is uniquely positioned to integrate signals derived from microbial components, such as lipopolysaccharide (LPS), as well as endogenous stressors including mitochondrial dysfunction, oxidative stress, ATP release, and metabolic dysregulation [7]. Upon activation, NLRP3 assembles a multiprotein complex that leads to caspase-1 activation and the subsequent maturation and release of the highly pro-inflammatory cytokines interleukin-1β (IL-1β) and interleukin-18 (IL-18), which exert profound effects on immune cell recruitment, epithelial barrier function, and tissue homeostasis [8,9].

The pathological relevance of NLRP3 inflammasome activation is supported by extensive evidence across multiple disease contexts. In metabolic disorders, NLRP3-driven IL-1β signaling contributes to insulin resistance, adipose tissue inflammation, and β-cell dysfunction [10]. In IBD, excessive inflammasome activation disrupts epithelial integrity and amplifies mucosal immune responses [11]. In neurodegenerative diseases, NLRP3 activation in microglia promotes neuroinflammation and accelerates neuronal damage, while in cardiovascular disease (CVD), it contributes to endothelial dysfunction and atherosclerotic plaque instability [12,13]. These observations highlight the NLRP3 inflammasome as a central molecular hub linking immune activation, metabolic stress, and tissue pathology.

Importantly, the gut represents a primary site for NLRP3 regulation, as it is continuously exposed to a vast array of microbial products and dietary components. Increased intestinal permeability, dysbiosis, and metabolic endotoxemia provide a constant source of priming and activation signals for inflammasome assembly, thereby coupling gut microbial ecology to systemic inflammatory tone [14,15]. This positioning of NLRP3 at the interface between the microbiota, host metabolism, and immune responses makes it a particularly attractive target for nutritional and microbiota-based interventions aimed at controlling chronic inflammation.

Diet represents one of the most powerful and dynamic environmental modulators of both gut microbiota composition and host inflammatory tone. Long-term dietary patterns shape the ecological structure and functional capacity of the intestinal microbiome, thereby influencing the production of microbial metabolites that directly interact with immune, metabolic, and epithelial cells [16]. Diets rich in fiber and plant-derived bioactive compounds promote a microbiota characterized by high diversity and metabolic flexibility, whereas Western-style diets, high in saturated fat and refined carbohydrates, are associated with dysbiosis, increased intestinal permeability, and chronic low-grade inflammation [17]. Through these mechanisms, diet acts not merely as a source of nutrients but as a primary regulator of host–microbe interactions and immune homeostasis.

Among dietary bioactives, polyphenols have attracted particular interest due to their consistent association with reduced incidence of metabolic, cardiovascular, and inflammatory diseases [18]. Epidemiological and interventional studies have repeatedly linked high polyphenol intake with improvements in inflammatory biomarkers, endothelial function, insulin sensitivity, and gut barrier integrity [19]. However, these beneficial effects cannot be fully explained by the classical paradigm that attributes polyphenol bioactivity to their direct antioxidant properties. In fact, most polyphenols exhibit low intestinal absorption, are rapidly conjugated in the liver, and circulate at concentrations that are often insufficient to exert direct systemic antioxidant or anti-inflammatory effects [20].

This apparent paradox has led to a paradigm shift in the understanding of polyphenol biology. It is now widely recognized that the gut microbiota acts as a metabolic interface that transforms dietary polyphenols into a broad array of low-molecular-weight metabolites with enhanced bioavailability, stability, and biological potency [21]. Through enzymatic processes such as de-glycosylation, ring fission, dehydroxylation, and reduction, intestinal bacteria convert complex polyphenols into compounds such as urolithins, phenyl-γ-valerolactones, and phenolic acids, which can readily cross the intestinal barrier and reach systemic circulation [22]. These microbial-derived metabolites interact with host cells through multiple molecular targets, including nuclear receptors, redox-sensitive transcription factors, and immune signaling pathways [23].

Importantly, an increasing body of evidence indicates that these microbial metabolites, rather than their parent polyphenols, are the primary mediators of the anti-inflammatory and cytoprotective effects attributed to polyphenol-rich diets. Many of these compounds have been shown to modulate key inflammatory pathways, including the nuclear factor kappa-light-chain-enhancer of activated B cells (NF-κB), nuclear factor erythroid 2-related factor 2 (Nrf2), and aryl hydrocarbon receptor signaling, as well as mitochondrial function and oxidative stress [24,25,26]. Of particular relevance to chronic inflammatory diseases is their emerging role in regulating inflammasome activation, especially the NLRP3 inflammasome, which integrates microbial, metabolic, and stress-related signals into the production of IL-1β and IL-18.

In this context, the gut microbiota emerges not as a passive recipient of dietary polyphenols, but as an active biochemical reactor that critically shapes their immunological impact. Interindividual differences in microbial composition and metabolic capacity generate substantial variability in polyphenol biotransformation, thereby influencing inflammatory susceptibility and disease risk [27]. Within this framework, the microbiota–polyphenol–NLRP3 inflammasome axis represents a central regulatory pathway through which dietary patterns are transduced into modulation of innate immune homeostasis, tissue integrity, and cellular resilience.

This review proposes that this axis constitutes a mechanistically coherent network linking diet to inflammation. Its understanding requires the integration of three interdependent levels: (i) the NLRP3 inflammasome as a convergent signalling hub for microbial, metabolic, and redox cues; (ii) the gut microbiota as an active metabolic intermediary that determines whether dietary polyphenols exert pro- or anti-inflammatory effects; and (iii) the emerging evidence that microbial-derived metabolites, rather than parent polyphenols, are the principal bioactive effectors of inflammasome modulation at both local and systemic levels. Critically, these three layers do not operate in isolation. The NLRP3 inflammasome responds to priming signals generated by dysbiosis, but inflammasome activation itself reshapes microbiota composition through IL-1β-driven mucosal inflammation, thus forming a bidirectional regulatory loop. Polyphenol biotransformation by gut bacteria interrupts this loop at multiple nodes simultaneously, suppressing NF-κB-mediated priming, enhancing mitophagy, and restoring barrier integrity, rather than acting on a single molecular target. It is this multi-node, self-reinforcing architecture that confers on the axis the properties of a network rather than a linear cascade, and that explains why its modulation through diet can produce system-level anti-inflammatory effects disproportionate to the action of any individual compound.

Characterising the interactions between these layers across experimental models and human studies provides the conceptual foundation for developing precision nutrition strategies and microbiota-targeted interventions aimed at preventing and treating chronic inflammatory disorders.

## 2. The NLRP3 Inflammasome as a Hub of Inflammation

**Figure 1:**
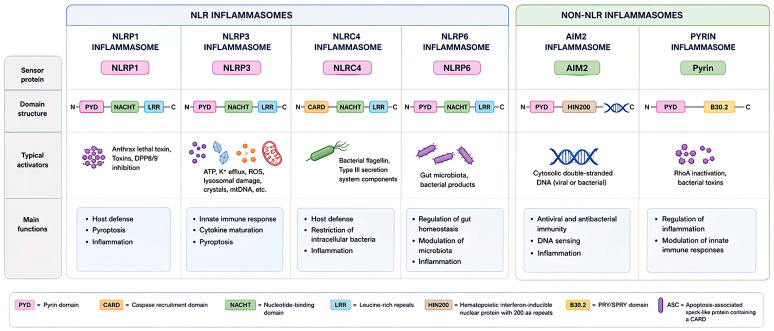
Schematic representation of the different types of inflammasome complexes.

Inflammasomes are cytosolic multiprotein complexes that play a central role in innate immunity by sensing PAMPs and DAMPs. Upon activation, these platforms orchestrate inflammatory responses primarily through the activation of caspase-1 and subsequent maturation of pro-inflammatory cytokines. Several types of inflammasomes have been described, each defined by a distinct sensor protein [28,29]. These include NLR family members such as NLRP1, NLRC4, and NLfRP6, as well as non-NLR sensors like AIM2 and pyrin, which respond to diverse microbial, environmental, and endogenous stress signals [30,31]. Despite their structural and functional diversity, all inflammasomes share a common mechanism involving the recruitment of the adaptor protein ASC and the activation of pro-caspase-1, ultimately leading to cytokine release and, in many cases, pyroptotic cell death (Figure 1).

The NLRP3 inflammasome is a cytosolic multiprotein complex that serves as one of the most versatile and sensitive innate immune sensors of cellular stress and danger [32]. It is composed of the pattern-recognition receptor NLRP3, the adaptor protein apoptosis-associated speck-like protein containing a CARD (ASC), and the effector enzyme pro-caspase-1. In resting conditions, NLRP3 is maintained in an auto-inhibited conformation, spatially separated from ASC and pro-caspase-1. Upon activation, NLRP3 undergoes conformational rearrangements that allow its oligomerization and recruitment of ASC, which in turn nucleates the formation of large supramolecular complexes known as ASC specks [33]. These platforms facilitate proximity-induced cleavage of pro-caspase-1 into its active form, triggering the proteolytic maturation of pro-IL-1β and pro-IL-18 and the cleavage of gasdermin D, leading to pyroptotic cell death and amplification of inflammatory signaling [34,35].

NLRP3 activation is governed by a tightly regulated two-step process that ensures inflammasome assembly occurs only in the presence of both inflammatory priming and cellular danger. The first signal, termed priming, is mediated by the engagement of pattern-recognition receptors such as Toll-like receptors or cytokine receptors, leading to activation of NF-κB and other transcription factors. This step results in increased transcription of NLRP3 itself as well as of pro-IL-1β and other inflammasome components, but also induces critical post-translational modifications, including ubiquitination, phosphorylation, and de-ubiquitination, that license NLRP3 for activation. In physiological conditions, this priming phase ensures that inflammasome activation is restricted to contexts in which immune activation is required [36,37].

The second signal, the activation step, is triggered by a wide range of DAMPs that reflect cellular dysfunction rather than specific microbial ligands. These include extracellular ATP released from damaged or stressed cells, potassium efflux through P2X7 receptors, calcium influx, lysosomal rupture induced by crystalline or particulate matter, and, critically, mitochondrial dysfunction [38]. Mitochondria act as central hubs for inflammasome activation, as they generate reactive oxygen species (ROS), release oxidized mitochondrial DNA, and provide a physical platform for NLRP3 assembly. These mitochondrial danger signals converge to destabilize cellular homeostasis and promote NLRP3 oligomerization, thereby coupling metabolic and redox stress directly to innate immune activation [39].

The gastrointestinal tract is uniquely positioned as a major site of NLRP3 inflammasome regulation. The intestinal mucosa is continuously exposed to an immense and dynamic burden of microbial products, dietary antigens, xenobiotics, and host-derived metabolites, all of which can influence both the priming and activation of inflammasomes. Under homeostatic conditions, a tightly regulated epithelial barrier, mucus layer, and immune network limit the translocation of microbial ligands and ensure that NLRP3 activation contributes to host defense and tissue repair. Indeed, physiological inflammasome signaling plays an important role in maintaining epithelial integrity, promoting antimicrobial peptide production, and shaping adaptive immune responses [40,41].

However, disruption of intestinal homeostasis profoundly alters this balance. Dysbiosis, dietary imbalance, antibiotic exposure, and chronic stress compromise epithelial tight junctions and mucus integrity, leading to increased intestinal permeability and metabolic endotoxemia [42]. This results in persistent exposure of mucosal and systemic immune cells to microbial-derived ligands such as LPS, peptidoglycans, and bacterial DNA, which continuously engage pattern-recognition receptors and sustain NF-κB-mediated priming of the NLRP3 inflammasome [43]. In parallel, microbial dysbiosis and Western-style diets promote mitochondrial dysfunction, oxidative stress, and altered cellular metabolism in epithelial and immune cells, providing a constant source of inflammasome-activating signals [44].

As a result, the gut becomes a chronic driver of systemic inflammasome activation. Low-grade endotoxemia originating from the intestine propagates NLRP3 signaling in metabolically active tissues such as adipose tissue, liver, pancreas, vascular endothelium, and even the central nervous system [45]. In adipose tissue, NLRP3 activation promotes macrophage infiltration and insulin resistance; in the liver, it drives steatohepatitis; in the brain, it sustains microglial activation and neuroinflammation; and in the vasculature, it contributes to endothelial dysfunction and atherosclerotic plaque instability [46]. Thus, intestinal dysregulation of NLRP3 links gut microbial ecology directly to the systemic inflammatory tone that underlies many chronic diseases.

While transient activation of the NLRP3 inflammasome is essential for antimicrobial defense and tissue repair, its chronic or inappropriate activation creates a self-perpetuating cycle of inflammation, mitochondrial dysfunction, and tissue damage. This pathological loop not only sustains the production of IL-1β and IL-18 but also amplifies oxidative stress, disrupts metabolic homeostasis, and compromises epithelial and endothelial barriers, further increasing exposure to microbial and danger signals [47]. This strategic positioning makes NLRP3 the convergence node of the axis: it receives priming inputs from dysbiosis-derived LPS and metabolic endotoxemia, is suppressed by polyphenol-derived microbial metabolites at both the priming and activation steps, and, once activated, generates inflammatory outputs that feed back to disrupt gut ecology, a feedback mechanism with direct pathological consequences across organ systems.

## 3. Gut Microbiota as a Regulator of Inflammasome Activity

The human body harbors trillions of microorganisms that collectively constitute the gut microbiome. Although each individual possesses a unique microbial composition, analogous to a biological fingerprint, a core microbiota shared across the human population can be identified, accounting for approximately 10% of the total microbial community [16]. An additional ~30% is shaped by host genetics and developmental factors, including sex, mode of delivery (vaginal birth or Caesarean section), geographical location, and age [48]. The remaining fraction of the gut microbiome is predominantly influenced by environmental and lifestyle factors, particularly dietary patterns and physical activity, highlighting its dynamic and adaptable nature. Far from being passive commensals, gut-associated bacteria, viruses, and fungi play essential roles in host metabolism, immune regulation, and neurobehavioral processes. In recent years, accumulating evidence has underscored the profound impact of the gut microbiome on human health, fostering new insights into nutrition, disease prevention, and personalized medicine [49].

**Figure 2:**
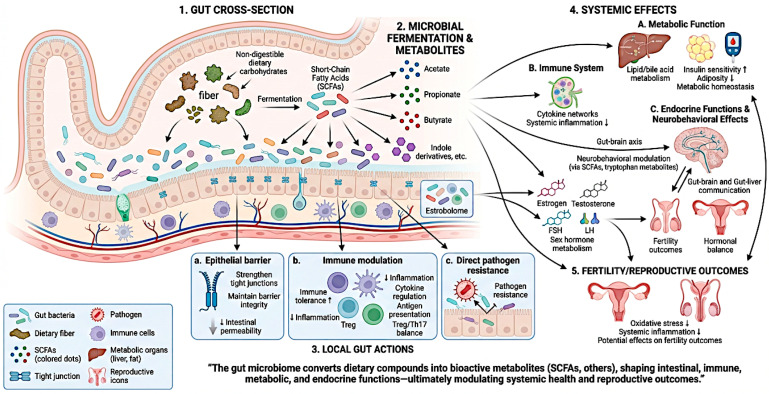
Schematic representation of the metabolic, immune, and endocrine functions of the gut microbiome. The gut microbiome regulates host metabolism, immune responses, and hormonal balance through the production of bioactive metabolites, interactions with the gut–brain and gut–liver axes, and modulation of sex hormone metabolism, thereby influencing metabolic and reproductive health.

The gut microbiome plays a pivotal role in host physiology, exerting wide-ranging effects on metabolic, immune, and endocrine functions (Figure 2) [50,51,52]. One of its primary roles is the fermentation of non-digestible dietary components, particularly complex carbohydrates, leading to the production of short-chain fatty acids (SCFAs), such as acetate, propionate, and butyrate, which contribute to energy homeostasis and intestinal barrier integrity, regulate gene expression, modulate inflammatory responses and affect neurobehavioral functions through receptor-mediated signaling and epigenetic mechanisms [53,54]. The gut microbiota is also essential for the development and regulation of the immune system, promoting immune tolerance while preventing excessive inflammatory activation and modulating antigen-specific responses, co-stimulatory signaling, cytokine production, and immune cell polarization, while also influencing pathogen resistance through indirect host-mediated mechanisms and direct microbial competition [55,56]. Furthermore, it supports the maintenance of gut barrier function by strengthening epithelial tight junctions and limiting increased intestinal permeability, thereby reducing systemic exposure to microbial-derived pro-inflammatory molecules [57]. Beyond local intestinal effects, the gut microbiome influences systemic metabolic health through its involvement in lipid and bile acid metabolism, regulation of insulin sensitivity, and modulation of adipose tissue accumulation [58,59]. Importantly, emerging evidence highlights the role of the gut microbiome in endocrine regulation via interactions with the gut–brain and gut–liver axes, as well as through the modulation of sex hormone metabolism by the estrobolome [60]. Through the production of bioactive metabolites, such as SCFAs and tryptophan-derived compounds, the gut microbiota exerts indirect effects on reproductive health by influencing hormonal balance, oxidative stress, and systemic inflammation, thereby potentially affecting male and female fertility outcomes. Collectively, these processes underscore the gut microbiome as an active, multifunctional regulator of host health, with profound implications for immunity, metabolism, and disease susceptibility.

Alterations in the composition of the bacterial microbiota, commonly referred to as dysbiosis, are widely recognized as a major contributing factor to a range of human diseases, including inflammatory disorders [61]. Across disease contexts, the crosstalk between gut microbiota and NLRP3 inflammasome activity converges on three interconnected mechanisms. First, dysbiosis promotes the translocation of microbial-derived ligands, particularly LPS, peptidoglycans, and bacterial DNA, into the systemic circulation, providing a sustained source of NF-κB-mediated priming signals that license NLRP3 for activation. Second, the disruption of intestinal barrier integrity amplifies this process by increasing the permeability of the epithelial layer, thereby transforming a localized microbial imbalance into systemic endotoxemia that reaches metabolically active distant organs. Third, dysbiosis depletes the microbial taxa responsible for producing protective metabolites, particularly short-chain fatty acids and secondary bile acids, whose physiological role includes the suppression of inflammasome priming and activation. Together, these three mechanisms generate a self-amplifying loop in which gut microbial perturbation drives NLRP3-mediated inflammation across multiple organ systems, as illustrated by the following evidence.

Across neurodegeneration, atherosclerosis, age-related atrial fibrillation, and alcohol-induced neuroinflammation, a shared upstream mechanism is evident: gut barrier disruption enables microbial LPS and peptidoglycans to reach systemic circulation, providing sustained NF-κB-mediated priming of NLRP3 in organs anatomically distant from the gut. In the central nervous system, gut-derived LPS and neuroinflammatory mediators converge on microglial NLRP3 activation, amplifying neurodegenerative cascades [14,62,63]. In the heart, aged-gut microbiota transplantation is sufficient to transfer AF susceptibility to young hosts, and NLRP3 inhibition attenuates atrial fibrosis, establishing a causal gut–heart axis [64]. In atherosclerosis, dysbiosis-driven vascular NLRP3 activation promotes endothelial dysfunction and plaque progression [65].

A second conserved mechanism involves the loss of microbiota-derived protective signals rather than the gain of pro-inflammatory ones. In IgA nephropathy, selective depletion of *Bifidobacterium* correlates with disease severity, and probiotic restoration of this taxon attenuates renal NLRP3/ASC/caspase-1 activation through SCFA-mediated pathways [66]. In hyperuricaemia-associated renal injury, reduced secondary bile acids, particularly deoxycholic acid, impair TGR5-cAMP-PKA signalling in tubular cells, unleashing NLRP3-dependent fibrosis [15]. In *S. aureus*-induced mastitis, restoration of DCA or *Clostridium scindens* reconstitutes TGR5-mediated NLRP3 suppression [67]. Together, these examples highlight depletion of protective taxa as equally important as enrichment of pro-inflammatory ones in determining inflammasome tone.

A critical insight emerging from these disease models is that the relationship between dysbiosis and NLRP3 is bidirectional. In acute pancreatitis, NLRP3 deficiency reshapes baseline microbiota composition and prevents overgrowth of pro-inflammatory taxa such as *Escherichia–Shigella*, while fecal microbiota transplantation reinstates inflammasome activation and exacerbates disease severity [68]. In MASLD, NLRP3-driven hepatic inflammation further compromises intestinal barrier function, creating a self-amplifying gut–liver loop. This bidirectionality distinguishes a network from a cascade: intervention at any node, whether through polyphenol-driven microbiota remodelling, direct NLRP3 inhibition, or barrier restoration, has the potential to interrupt the loop rather than merely dampening a single output.

An important source of interpretive complexity that warrants explicit discussion is the context-dependent and at times conflicting role of NLRP3 in intestinal homeostasis. While the evidence reviewed above emphasises the pathological consequences of dysregulated NLRP3 activation, it is equally established that physiological inflammasome signalling is required for epithelial repair, antimicrobial peptide secretion, and the maintenance of mucosal immune balance. Studies in NLRP3-deficient mice have produced both protective and detrimental outcomes depending on the inflammatory model, the genetic background of the host, and the specific microbial community present, demonstrating that the consequences of NLRP3 loss-of-function are not uniform across disease contexts. This ambiguity is particularly pronounced in IBD, where the same inflammasome pathway can contribute to either barrier protection or pathological inflammation depending on the cellular compartment, epithelial versus immune, the activating stimulus, and the phase of disease. These conflicting findings preclude simple interpretations of NLRP3 suppression as uniformly beneficial and caution against direct extrapolation of findings from models of acute NLRP3 hyperactivation to the chronic, relapsing-remitting inflammatory contexts that characterise most human diseases.

While these findings support an association between gut microbiota alterations and NLRP3 inflammasome activation across different disease contexts, the evidentiary hierarchy underlying these claims varies substantially and must be interpreted with care. The strongest experimental support for causality derives from germ-free and fecal microbiota transplantation studies in rodents, which demonstrate that microbial communities are necessary and sufficient to modulate inflammasome activity in the host. However, these models involve microbial communities that differ substantially from human gut microbiota in taxonomic composition, metabolic capacity, and immune context, limiting direct cross-species inference. At the clinical level, the relationship between dysbiosis and NLRP3 activity is primarily established through association studies in patients, which cannot exclude confounding by disease severity, pharmacological treatment, or dietary habits independent of microbiota composition. Direct measurement of NLRP3 inflammasome activity in human tissues is methodologically unfeasible in most clinical settings, and surrogate endpoints such as circulating IL-1β and IL-18, while informative, lack the mechanistic specificity required to attribute inflammatory outputs unambiguously to caspase-1-dependent inflammasome processing rather than to alternative inflammatory pathways. These distinctions between associative and causal evidence are not merely academic: they define the boundaries of what can currently be concluded about the gut microbiota–NLRP3 axis in humans and underscore the need for mechanistically rigorous human studies as a priority for the field.

An important but often underappreciated dimension of gut microbiota–NLRP3 crosstalk is the degree to which its inflammatory consequences depend on the specific compositional configuration of the microbial community. Western-type microbiota, characterized by reduced diversity, depletion of *Faecalibacterium prausnitzii*, *Akkermansia muciniphila*, and *Bifidobacterium* spp., and relative enrichment of *Proteobacteria* and LPS-producing Gram-negative species, creates a pro-inflammatory baseline that chronically primes the NLRP3 inflammasome through sustained endotoxemia and reduced SCFA production. In contrast, microbiota configurations enriched in butyrate-producing *Firmicutes* and mucin-degrading mucosal commensals maintain barrier integrity and generate suppressive metabolic signals that hold inflammasome activation in check. Critically, these compositional differences determine not only the baseline inflammatory tone but also the individual’s capacity to respond to dietary polyphenol interventions: the same polyphenol-rich diet will generate profoundly different metabolite profiles and anti-inflammatory outcomes depending on whether the host microbiota contains the enzymatic repertoire needed for biotransformation. This compositional dependency is one of the principal reasons why polyphenol interventions produce heterogeneous results across clinical populations and why microbiota characterization at baseline should be considered a standard covariate in future nutritional intervention trials (Table 1).

## 4. Polyphenols

Polyphenols represent one of the most abundant groups of bioactive compounds present in plant-based foods and beverages, and they constitute a major class of dietary phytochemicals implicated in the prevention of chronic diseases [20]. Chemically, polyphenols are characterized by the presence of one or more phenolic rings bearing hydroxyl substituents and are typically classified into several subclasses based on structural complexity, including flavonoids (flavonols, flavones, flavan-3-ols, flavanones, isoflavones, and anthocyanins), phenolic acids (hydroxybenzoic and hydroxycinnamic acids), stilbenes, lignans, and high-molecular-weight tannins [69]. These compounds are widely distributed in fruits, vegetables, nuts, seeds, legumes, whole grains, tea, coffee, cocoa, herbs, spices, and red wine, contributing not only to sensory properties such as color, bitterness, and astringency but also to biological functions relevant to human health [70].

Over the past decades, accumulating epidemiological evidence has consistently associated high consumption of polyphenol-rich diets with reduced incidence of CVD, metabolic disorders, neurodegenerative conditions, and certain cancers [71,72,73]. Intervention studies further support their beneficial effects on endothelial function, insulin sensitivity, lipid metabolism, blood pressure regulation, cognitive performance, and inflammatory biomarkers [74,75,76]. These health-promoting effects have traditionally been attributed to the antioxidant capacity of polyphenols; however, it is now widely recognized that their biological activity extends far beyond direct radical scavenging. Polyphenols function as signaling molecules capable of modulating intracellular pathways involved in inflammation, oxidative stress, mitochondrial function, apoptosis, and cellular metabolism [77,78,79].

At habitual dietary intake levels, polyphenols consumed through whole foods show a favorable safety profile with no documented adverse effects. At supplemental doses, however, safety concerns emerge: high-dose resveratrol (≥1 g/day) may interfere with CYP3A4-dependent drug metabolism; curcumin has been associated with hepatotoxic potential in predisposed individuals; quercetin may interact with certain chemotherapeutics. These considerations are particularly relevant for elderly individuals and patients on polypharmacy regimens. A fundamental challenge remains the gap between concentrations used in preclinical studies and those achievable through diet or standard supplementation, which reinforces the role of microbial metabolites as primary bioactive effectors. The populations most likely to benefit are those with established metabolic risk factors and elevated baseline inflammatory tone, where NLRP3 overactivation provides a detectable therapeutic target.

**Figure 3:**
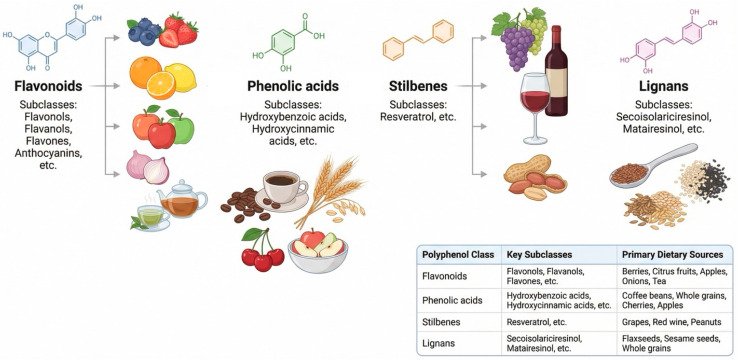
Main categories of polyphenols and their primary dietary sources.

Their pleiotropic nature allows them to influence multiple cellular targets simultaneously, positioning them as important dietary modulators of immune and metabolic homeostasis (Figure 3).

### Polyphenols as Direct Modulators of NLRP3 Signaling

Chronic inflammation represents a key pathogenic mechanism underlying many of the diseases in which polyphenols show protective effects. Consequently, increasing attention has focused on the ability of polyphenols to modulate innate immune signaling pathways, particularly the NLRP3 inflammasome [80].

One of the most consistently reported mechanisms involves suppression of the priming signal mediated by NF-κB. Polyphenols such as resveratrol, curcumin, quercetin, epigallocatechin gallate (EGCG), and luteolin inhibit upstream kinases including IKK and MAPKs, preventing nuclear translocation of NF-κB and reducing transcription of inflammasome-related genes. In parallel, polyphenols modulate epigenetic regulators and transcriptional coactivators that further influence inflammatory gene expression, suggesting that they act at multiple regulatory levels during the priming phase [81,82].

In addition to transcriptional control, polyphenols strongly influence the cellular stress signals that initiate inflammasome activation. Oxidative stress is a major driver of NLRP3 assembly, and many polyphenols activate the Nrf2 pathway, which enhances endogenous antioxidant defenses and maintains redox homeostasis. By limiting mitochondrial ROS production and oxidative injury, polyphenols disrupt a critical upstream trigger of inflammasome activation [83,84].

Mitochondrial function itself represents a central target of polyphenol action. Mitochondrial dysfunction not only generates ROS but also releases mitochondrial DNA and cardiolipin, which act as direct inflammasome activators. Several polyphenols enhance mitochondrial biogenesis, improve respiratory efficiency, and stimulate mitophagy through activation of AMP-activated protein kinase (AMPK), sirtuins, and peroxisome proliferator-activated receptor gamma coactivator-1α (PGC-1α) [39]. Resveratrol is a prominent example, as it activates SIRT1-AMPK signaling, enhances mitochondrial turnover, and suppresses inflammasome activation in metabolic and inflammatory models [85].

Polyphenols also influence ionic fluxes that are essential for inflammasome activation [86]. Potassium efflux is considered a common event required for NLRP3 activation, and certain polyphenols have been shown to modulate ion channels and membrane transporters, stabilizing intracellular ionic balance and preventing inflammasome assembly. Similarly, calcium signaling and lysosomal integrity are affected by polyphenols, which can stabilize lysosomal membranes and prevent the release of cathepsins into the cytosol, another known trigger of NLRP3 activation [87,88].

Another layer of regulation involves autophagy, a cellular process that removes damaged organelles and protein aggregates. Impaired autophagy enhances inflammasome activation by allowing accumulation of dysfunctional mitochondria and inflammasome components. Many polyphenols stimulate autophagic pathways through AMPK activation and mTOR inhibition, thereby indirectly suppressing NLRP3 activation. Enhanced autophagy contributes not only to reduced inflammasome signaling but also to improved cellular resilience under stress conditions [89].

Beyond intracellular mechanisms, polyphenols exert important immunomodulatory effects on innate immune cells. They promote macrophage polarization toward anti-inflammatory phenotypes, inhibit pro-inflammatory cytokine production, and modulate dendritic cell maturation and T-cell responses. These immune-regulatory actions create a cellular environment less permissive to inflammasome activation. Moreover, polyphenols improve endothelial function and epithelial barrier integrity, reducing exposure to circulating danger signals that contribute to systemic inflammasome activation [90].

Despite these direct mechanisms, an important consideration is that many experimental studies employ concentrations of native polyphenols that exceed those typically achieved through dietary intake. This discrepancy has led to increasing recognition that polyphenol metabolites generated by host and microbial metabolism may contribute substantially to their biological effects in vivo. Nevertheless, the ability of parent polyphenols to directly target multiple steps of the NLRP3 signaling cascade, including NF-κB activation, oxidative stress, mitochondrial dysfunction, ion fluxes, and autophagy, provides a strong mechanistic basis for their anti-inflammatory properties [23].

It is important to note, however, that the mechanisms described above are not uniformly shared across polyphenol subclasses. Stilbenes such as resveratrol exert their predominant effects through SIRT1-AMPK activation and mitochondrial biogenesis, with relatively modest direct impact on NF-κB-dependent priming compared to flavonols. Flavanols, particularly EGCG, are distinguished by their capacity to directly chelate metals involved in ROS generation and to modulate autophagy flux through mTOR inhibition, effects less documented for anthocyanins or isoflavones. Curcuminoids, belonging to the diarylheptanoid class, are notable for their broad upstream NF-κB suppression and their capacity to interfere with ASC oligomerization, but are severely limited by their intrinsic chemical instability and extremely poor systemic bioavailability, making their in vivo relevance highly dependent on formulation strategies or microbial conversion. Anthocyanins, despite their abundance in the diet, are rapidly degraded in the gastrointestinal tract and exert their anti-inflammatory effects predominantly through their phenolic acid degradation products rather than through the parent glycoside. Isoflavones, which are structurally similar to estrogens, modulate NLRP3 signaling partly through estrogen receptor-mediated pathways, introducing a degree of sex-specificity in their effects not observed in other subclasses. These distinctions are not merely academic: they have direct implications for the design of dietary interventions, the interpretation of clinical trials, and the identification of which polyphenol subclasses are most relevant for specific disease contexts.

**Figure 4:**
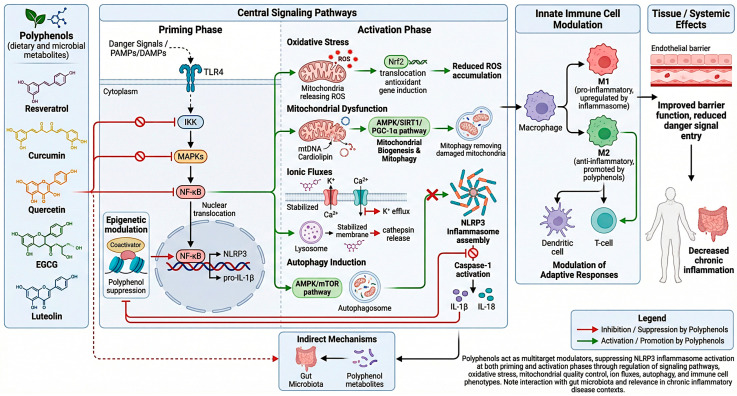
Schematic representation of the various mechanisms by which different types of polyphenols regulate the NLRP3 complex.

Collectively, these observations support the concept that dietary polyphenols act as multitarget modulators of innate immune signaling, capable of attenuating inflammasome activation through coordinated regulation of cellular metabolism, redox balance, and immune responses. This multifunctional activity is particularly relevant in the context of chronic inflammatory diseases characterized by persistent NLRP3 activation. Importantly, the direct effects of polyphenols on inflammasome pathways likely interact with indirect mechanisms mediated by gut microbiota modulation and microbial metabolite production, which together shape the overall impact of polyphenol-rich diets on inflammation and cellular protection [21] (Figure 4).

Polyphenols also exert indirect anti-inflammatory effects through modulation of gut microbiota composition and function. A large fraction of dietary polyphenols reaches the colon, where they are biotransformed by microbial enzymes into smaller phenolic acids and other metabolites with enhanced bioavailability and bioactivity [91]. In turn, polyphenols act as prebiotic-like compounds, selectively promoting the growth of beneficial taxa such as *Bifidobacterium* and *Akkermansia* while suppressing pathobionts associated with dysbiosis. These shifts in microbial ecology are accompanied by increased production of SCFAs, including butyrate, which strengthen intestinal barrier integrity and exert systemic anti-inflammatory effects [92]. Furthermore, polyphenol-derived metabolites can modulate microbial signaling and host–microbe interactions, influencing Toll-like receptor activity and reducing endotoxin translocation. Through these microbiota-dependent mechanisms, polyphenols indirectly attenuate NLRP3 inflammasome activation, linking dietary intake to systemic immune regulation.

However, the translational relevance of these findings is limited by the fact that many studies employ concentrations of polyphenols that are not achievable through dietary intake. In addition, most experiments are conducted in simplified in vitro systems that do not account for the extensive metabolic transformation of polyphenols occurring in vivo. As a result, the direct inhibitory effects on NLRP3 inflammasome activation may not fully reflect physiological conditions.

Importantly, the distinction between direct and indirect polyphenol effects is not merely academic: it reflects the two complementary nodes through which polyphenols engage the axis. The direct mechanisms modulate NLRP3 primarily at the priming and activation steps; the indirect, microbiota-dependent mechanisms generate bioactive catabolites that act on the same targets but at physiologically relevant concentrations and with superior systemic bioavailability. The integration of these two nodes is what makes the microbiota–polyphenol–NLRP3 axis a genuine regulatory network rather than a collection of parallel pathways.

## 5. Microbial Metabolites of Polyphenols as Inflammasome Regulators

A growing body of evidence, as described before, indicates that the biological effects of dietary polyphenols on inflammation cannot be fully understood without considering the metabolic contribution of the gut microbiota. Indeed, most native polyphenols reach the colon largely unmetabolized due to their complex structures, limited absorption, and extensive phase II metabolism in enterocytes and hepatocytes. In the colon, microbial enzymatic activities, including deglycosylation, dehydroxylation, demethylation, ring fission, and reduction, generate a wide spectrum of low-molecular-weight phenolic metabolites that display enhanced bioavailability and biological activity compared with their parent compounds [22]. These microbial-derived metabolites have emerged as critical mediators of host–microbiota communication and are increasingly recognized as potent modulators of innate immune signaling pathways, including the NLRP3 inflammasome.

Importantly, interindividual variability in microbiota composition determines the metabolic fate of polyphenols, giving rise to the concept of “metabotypes.” For example, the conversion of ellagitannins into urolithins depends on the presence of specific bacterial taxa such as *Gordonibacter* spp., leading to heterogeneous systemic exposure among individuals. This variability has profound implications for the anti-inflammatory efficacy of polyphenol-rich diets and may partly explain inconsistent outcomes observed in clinical studies [93].

Among the diverse metabolites generated by gut bacteria, several classes have demonstrated the capacity to modulate inflammasome signaling. Urolithins, particularly urolithin A (UA), represent one of the most extensively studied microbial polyphenol metabolites. These compounds are generated from ellagic acid present in pomegranate, berries, and nuts. Experimental evidence strongly supports their role in inflammasome modulation. For instance, Chen et al. demonstrated that UA significantly inhibited NLRP3 inflammasome activation in LPS-primed microglial cells, reducing caspase-1 cleavage, IL-1β secretion, and pyroptosis while promoting mitophagy and mitochondrial function [94].

Similarly, recent work by Peng Chen et al. showed that methylated UA improved cognitive function in aging mice by suppressing NLRP3 activation and restoring mitochondrial bioenergetics via the p53-PGC-1α pathway [95].

Similarly, the results shown by Qiu et al. provide strong evidence that UA protects against dopaminergic neurodegeneration and neuroinflammation. According to the authors, the main mechanism, demonstrating that UA reduces NLRP3 inflammasome activation both in vitro and in vivo, may be related precisely to the inhibition of NLRP3 inflammasome activation through the promotion of mitophagy in microglia [96].

In an elegant study in 2024, UA inhibited caspase-1 cleavage induced by the NLRP3 inflammasome agonist, IL-1β maturation, and pyroptosis activation in bone marrow-derived macrophages from mice. UA also reduced intracellular and mitochondrial ROS generation and limited the interaction between thioredoxin-interacting protein and NLRP3, attenuating NLRP3 inflammasome activation [97].

A study conducted by Cho et al. demonstrated the impact of UA and its combination with eicosapentaenoic acid on mitigating NLRP3 inflammasome activation. The results indicated that both eicosapentaenoic acid and UA had a positive effect in attenuating caspase-1 production, leading to a dramatic decrease in IL-1β secretion. In particular, according to the authors, enriching the diet with eicosapentaenoic acid and UA precursors may be an effective strategy for mitigating neuroinflammation and neurodegenerative diseases [98]. These findings highlight a central concept: microbial metabolites can modulate inflammasome activity indirectly through mitochondrial quality control mechanisms, including mitophagy, ROS reduction, and metabolic reprogramming. It should be noted, however, that the mechanistic evidence for urolithin A is not uniformly consistent across experimental systems. While promitophagic and NLRP3-suppressive effects are well established in microglial and macrophage models, evidence in hepatic and endothelial cell systems is more limited and, in some cases, shows attenuated or context-dependent responses, potentially reflecting cell-type-specific differences in mitophagy flux, NLRP3 assembly dynamics, or intracellular metabolite distribution. Furthermore, the dependence of urolithin A production on the presence of specific bacterial taxa, particularly *Gordonibacter* spp., means that a substantial proportion of the human population does not produce this metabolite at detectable systemic levels regardless of ellagitannin intake. This source of interindividual variability is inconsistently controlled for in clinical studies and has not yet been systematically addressed in the urolithin A intervention literature, limiting the generalisability of findings from studies conducted in metabotype-uncharacterised populations.

Flavonoids represent one of the most extensively studied subclasses of dietary polyphenols, largely due to their abundance in commonly consumed foods such as berries, cocoa, tea, apples, onions, and red wine. Despite their structural diversity, a common characteristic shared by most flavonoids, including catechins, anthocyanins, and flavonols, is their relatively low bioavailability in native form [99]. Following ingestion, only a small fraction is absorbed in the small intestine, while the majority reaches the colon, where it undergoes extensive biotransformation by the gut microbiota. This microbial metabolism generates a wide spectrum of low–molecular-weight compounds, notably phenyl-γ-valerolactones and various phenolic acids such as protocatechuic acid, gallic acid, and 3,4-dihydroxyphenylacetic acid. These metabolites differ substantially from their parent compounds in terms of physicochemical properties, systemic exposure, and biological activity, and they are increasingly recognized as the primary mediators of the health effects traditionally attributed to dietary flavonoids [100].

One of the most relevant features of these microbiota-derived metabolites is their markedly higher systemic bioavailability compared with the parent flavonoids. Their smaller molecular size and increased polarity facilitate intestinal absorption and systemic distribution, enabling them to reach peripheral tissues at physiologically relevant concentrations. Importantly, several of these metabolites have also demonstrated the capacity to cross biological barriers, including the blood–brain barrier, thereby extending their potential influence to the central nervous system. This pharmacokinetic profile is particularly relevant in the context of inflammasome biology, as NLRP3 activation occurs not only in peripheral immune cells but also in tissue-resident macrophages, endothelial cells, adipocytes, hepatocytes, and microglial cells. Consequently, microbial-derived phenolic metabolites are well-positioned to exert systemic immunomodulatory effects across multiple organs [101].

Protocatechuic acid provides a representative example of how microbial-derived metabolites modulate inflammasome signaling. Multiple experimental studies have demonstrated that this compound acts on both the priming and activation steps of the inflammasome. For instance, experimental models of metabolic inflammation have shown that protocatechuic acid decreases the expression of NLRP3, ASC, and caspase-1 while simultaneously lowering IL-1β secretion. Similar observations have been reported in macrophage and endothelial cell models, where protocatechuic acid attenuated inflammatory responses induced by LPS or metabolic stressors. These findings suggest that this metabolite interferes with both the priming and activation phases of inflammasome signaling, highlighting its potential as a multitarget modulator of inflammatory cascades [102,103].

Phenyl-γ-valerolactones, which are major microbial metabolites of flavan-3-ols such as epicatechin and catechin, have also attracted considerable interest due to their anti-inflammatory properties. Studies in macrophage models have demonstrated that these metabolites reduce inflammatory cytokine production by modulating upstream signaling pathways, particularly mitogen-activated protein kinases (MAPKs) and NF-κB. Although direct investigations on NLRP3 assembly remain limited, the upstream signaling effects observed strongly support their role as modulators of inflammasome-related inflammation [104].

Anthocyanin-derived metabolites constitute another important group of bioactive compounds generated through microbial metabolism. Parent anthocyanins are generally unstable under physiological conditions and exhibit low absorption, whereas their degradation products, including phenolic acids and conjugated derivatives, achieve significantly higher circulating levels. Experimental evidence indicates that these metabolites can regulate inflammasome activation in immune and neural cells. For example, Sood et al. demonstrated that metabolites derived from malvidin suppressed the activation of multiple inflammasome complexes, including NLRP3, NLRC4, and AIM2, in microglial cells. This effect was associated with reduced caspase-1 activation, decreased IL-1β release, and attenuation of neuroinflammatory responses. Such findings are particularly relevant in the context of neurodegenerative diseases, where chronic microglial inflammasome activation contributes to disease progression [105].

Collectively, these observations support the concept that flavonoid-derived microbial metabolites act as key signaling molecules linking diet, microbiota activity, and host immune regulation. Rather than functioning solely as antioxidants, these compounds operate as modulators of intracellular signaling networks that control inflammasome activation, mitochondrial homeostasis, and inflammatory gene expression. Their capacity to simultaneously influence multiple regulatory nodes, including NF-κB signaling, oxidative stress pathways, and mitochondrial function, positions them as central mediators within the microbiota–polyphenol–inflammasome axis. This paradigm shift underscores the importance of considering microbial metabolism when evaluating the biological effects of dietary polyphenols and provides a mechanistic framework for understanding how flavonoid-rich diets contribute to the prevention of chronic inflammatory diseases.

Although microbial-derived polyphenol metabolites are increasingly recognised as key bioactive mediators, direct evidence linking these compounds to NLRP3 modulation in humans remains scarce. Most available data are derived from in vitro experiments or animal models, and only a limited number of studies have quantified circulating or tissue-specific metabolite levels in relation to inflammasome activity. This gap represents a major obstacle for establishing causal and clinically relevant relationships. A further and underappreciated limitation concerns the concentration at which these metabolites exert their documented effects. The majority of mechanistic studies demonstrating NLRP3 suppression by urolithin A, protocatechuic acid, phenyl-γ-valerolactones, and anthocyanin-derived metabolites employ concentrations that are achievable only under supraphysiological supplementation conditions or that exceed those typically measured in plasma or target tissues following habitual dietary exposure. This discrepancy between experimental and physiological concentrations means that in vitro mechanistic findings, while providing valid proof-of-concept for molecular pathways, cannot be directly extrapolated to predict the magnitude or direction of effects at concentrations realistically achievable through diet (Table 2).

A cross-metabolite analysis of the evidence reviewed above reveals a mechanistic convergence that is itself a defining property of the axis. Despite arising from structurally unrelated polyphenol precursors through distinct microbial enzymatic routes, urolithin A, phenyl-γ-valerolactones, protocatechuic acid, and anthocyanin-derived phenolics all suppress NLRP3 through an overlapping set of molecular nodes: inhibition of NF-κB-dependent priming, enhancement of mitochondrial quality control (mitophagy, ROS reduction, respiratory efficiency), and, in several cases, direct interference with caspase-1 activation. This mechanistic redundancy is not coincidental; it reflects the capacity of the microbiota to generate multiple chemically distinct effectors that collectively ensure robust NLRP3 suppression even when a single metabolite pathway is absent or insufficient. This built-in redundancy is one of the features that make the microbiota–polyphenol interface a resilient regulatory network rather than a fragile single-pathway system.

### Polyphenols, Microbiota and Intestinal Barrier Integrity

A central mechanism through which polyphenols and their microbiota-derived metabolites contribute to inflammasome regulation involves the preservation of intestinal barrier integrity, which represents a critical interface between the host immune system and luminal microbial signals. The intestinal epithelium functions not only as a physical barrier but also as an immunologically active structure that continuously integrates microbial, dietary, and metabolic cues. Disruption of this barrier, often referred to as “leaky gut,” facilitates the translocation of bacterial components such as LPS into the circulation, thereby promoting chronic low-grade inflammation and sustained priming of the NLRP3 inflammasome. This process is particularly relevant in metabolic disorders, IBD, and cardiometabolic conditions, where increased intestinal permeability is recognized as a major pathogenic driver [106].

Polyphenol-rich diets have consistently been shown to promote a favorable remodeling of the gut microbiota, characterized by the enrichment of beneficial commensal species such as *Akkermansia muciniphila* and *Faecalibacterium prausnitzii* [19]. These bacteria play key roles in maintaining mucosal homeostasis by stimulating mucus layer production, enhancing tight junction protein expression, and producing metabolites that support epithelial energy metabolism. Experimental evidence indicates that polyphenol-induced increases in *Akkermansia muciniphila* abundance are associated with improved gut barrier function, reduced metabolic endotoxemia, and attenuation of systemic inflammation, as demonstrated for example by Roopchand et al., who reported that grape polyphenol supplementation promoted *Akkermansia* growth and improved metabolic parameters in obese mice [107]. Similarly, Anhê et al. showed that cranberry-derived polyphenols remodeled the gut microbiota and improved metabolic inflammation, effects closely linked to barrier restoration. Beyond microbial compositional changes, microbial metabolites generated from polyphenol biotransformation further contribute to epithelial protection by modulating oxidative stress, inflammatory signaling, and mitochondrial function within intestinal cells [108].

An additional and highly relevant layer of this barrier-centered mechanism involves SCFAs, including acetate, propionate, and butyrate. Although SCFAs are not direct metabolites of polyphenols, a growing body of evidence indicates that their production is frequently enhanced following the consumption of polyphenol-rich diets as a consequence of microbiota remodeling.

SCFAs exert potent anti-inflammatory effects through multiple molecular mechanisms that converge on immune regulation and cellular homeostasis. One of the most extensively characterized pathways involves the inhibition of histone deacetylases (HDAC), leading to epigenetic reprogramming of immune cells. Butyrate, in particular, is a strong HDAC inhibitor capable of modulating gene expression profiles associated with inflammatory responses, oxidative stress resistance, and cellular metabolism [109]. In addition to epigenetic regulation, SCFAs function as ligands for specific G-protein–coupled receptors (GPCR), including GPR41 (FFAR3), GPR43 (FFAR2), and GPR109A (HCAR2), which are expressed on intestinal epithelial cells, macrophages, dendritic cells, and other immune populations. Activation of these receptors triggers intracellular signaling cascades that promote anti-inflammatory cytokine production, enhance barrier function, and regulate immune cell differentiation [110].

Among SCFAs, butyrate has received particular attention due to its robust immunomodulatory properties and its capacity to influence adaptive immune responses. One of the hallmark effects of butyrate is the promotion of regulatory T cell (Treg) differentiation through epigenetic mechanisms involving HDAC inhibition and enhanced acetylation of the Foxp3 promoter region. Increased Treg populations contribute to immune tolerance and suppression of excessive inflammatory responses, thereby indirectly limiting inflammasome activation. Experimental evidence supports this concept. Smith et al. demonstrated that SCFAs derived from microbial fermentation promote the expansion of colonic Treg cells and protect against inflammatory diseases, highlighting a mechanistic link between microbial metabolites and immune homeostasis [111]. In addition, Mann et al. showed that butyrate induces differentiation of colonic regulatory T cells through epigenetic modulation, further supporting the immunoregulatory role of SCFAs [112].

More direct evidence regarding inflammasome regulation has also been reported. For example, Youm et al. demonstrated that the ketone body β-hydroxybutyrate inhibits NLRP3 inflammasome activation by preventing potassium efflux and ASC oligomerization, providing proof-of-concept that metabolites structurally related to butyrate can directly interfere with inflammasome assembly [113]. Subsequently, several studies specifically investigating SCFAs confirmed similar anti-inflammasome effects. Chang et al. showed that butyrate suppresses LPS-induced inflammatory responses in macrophages through HDAC inhibition and metabolic reprogramming, mechanisms closely linked to reduced inflammasome priming [114]. More recently, Yuan et al. reported that butyrate attenuates NLRP3 inflammasome activation in macrophages by inhibiting NF-κB signaling and reducing mitochondrial ROS production [115]. In models of intestinal inflammation, Macia et al. demonstrated that SCFAs protect against colitis through GPCR-dependent pathways that regulate immune responses and epithelial integrity, processes known to influence inflammasome activity [116].

It should be noted, however, that the observed increases in SCFA production following polyphenol-rich diets may not be exclusively attributable to polyphenol-driven microbiota modulation. In many dietary contexts, high polyphenol intake is accompanied by increased consumption of plant-derived foods that are also rich in fermentable dietary fibers, which themselves represent the primary substrates for microbial SCFA production [117]. Therefore, disentangling the specific contribution of polyphenols from that of dietary fiber is challenging in both clinical and nutritional intervention studies. Nonetheless, accumulating evidence from controlled experimental models using purified polyphenols supports a direct modulatory role of polyphenols on microbial composition and metabolic activity, suggesting that both intrinsic polyphenol effects and co-ingested dietary fibers likely act synergistically in shaping SCFA-mediated host responses.

Taken together, these findings support the concept that SCFAs act as crucial secondary mediators within the microbiota–polyphenol–host axis. By integrating epigenetic regulation, receptor-mediated signaling, immune cell differentiation, and barrier protection, SCFAs provide multiple layers of control over NLRP3 inflammasome activation (Table 3). This indirect pathway highlights the importance of considering not only the direct microbial metabolites of polyphenols but also the broader metabolic ecosystem reshaped by polyphenol consumption when evaluating their anti-inflammatory and cytoprotective effects.

## 6. Relevance to Chronic Diseases

The most prevalent chronic diseases include heart disease, cancer, stroke, chronic lower respiratory diseases and Alzheimer’s disease, which are among the leading causes of morbidity and mortality worldwide and are expected to place a significant economic burden on the global economy. Additionally, the increase in life expectancy has contributed to a higher prevalence of chronic diseases, as longer lifespans allow for the accumulation of risk factors and age-related physiological changes [118]. Lifestyle behaviors and community factors (physical inactivity, unhealthy diets, tobacco and alcohol) significantly influence both the development and management of these conditions [119,120]. A common underlying factor among these conditions is inflammation, often accompanied by oxidative stress, which results from an imbalance between antioxidants and pro-oxidants.

The microbiota–polyphenol–NLRP3 axis, described above, carries direct pathophysiological relevance across several chronic disease domains in which sustained inflammasome activation constitutes a recognized driver of tissue injury and disease progression [121].

### 6.1. Cardiovascular Diseases

Growing evidence shows that polyphenols have protective effects against CVD by improving vascular function, inhibiting platelet aggregation, reducing inflammation, preventing LDL oxidation, and enhancing blood lipid profiles. Randomized trials also demonstrate their ability to lower blood pressure and reduce CVD-related mortality [122,123].

Dietary polyphenols, particularly resveratrol, EGCG, and quercetin, are inversely associated with CVD risk according to epidemiological and mechanistic evidence. At the molecular level, these compounds reduce oxidative stress by regulating oxidase activity and enhancing antioxidant enzyme expression, improve endothelial function through increased nitric oxide production and vascular relaxation, and protect against conditions such as stroke, hypertension, heart failure and ischemic heart disease [124]. Higher polyphenol intake has also been associated with lower blood pressure and reduced cardiovascular risk, as both dietary and metabolite-based polyphenol scores were inversely correlated with diastolic blood pressure, atherosclerotic CVD risk, and HeartScore, while positively influencing HDL cholesterol [125]. In addition, polyphenols inhibit platelet aggregation, reducing thrombosis and vascular dysfunction involved in AS, and exert anti-inflammatory and antithrombotic effects while improving lipid metabolism, including cholesterol and triglyceride levels [126].

Given the potential of polyphenols to reduce CVD risk through the modulation of inflammatory pathways such as the NLRP3 inflammasome, Villalva et al. conducted a comprehensive review of 18 in vivo studies. Their findings showed that dietary polyphenols consistently modulate the NLRP3 inflammasome pathway in cardiometabolic diseases, reducing NLRP3 expression and improving disease-related markers. Preclinical models included MCAO/R and MI/R in rats or mice, as well as ApoE^−/−^ atherosclerotic mice, ventricular arrhythmia in rabbits, anthracycline-induced cardiac damage, permanent coronary occlusion and hepatic ischemia/reperfusion. Tested polyphenols comprised flavonoids (flavonols, dihydroflavonols, flavones, flavanones, procyanidins), stilbenes (resveratrol), phenolic acids (salvianolic acids B, D, Y, rosmarinic acid), other phenolics (bakuchiol, carthamin yellow, 6-gingerol) and plant extracts (Abelmoschus manihot, Herba Siegesbeckiae, Rhodiola crenulate). At the molecular level, polyphenols downregulated NLRP3, ASC, and caspase-1, modulated upstream markers such as TLR4 and phosphorylated NF-κB, and reduced IL-1β and IL-18 in serum, often in a dose-dependent manner. These results indicate that polyphenols exert anti-inflammatory effects via NLRP3 inflammasome inhibition, supporting their potential role in preventing and mitigating chronic CVD [80]. Together, these mechanisms contribute to improved vascular function and reduced cardiovascular risk, supporting the role of polyphenol-rich diets in the prevention and management of CVD.

### 6.2. Metabolic, Endocrine and Digestive Diseases

Oxidative stress and inflammation play central roles in the development of chronic metabolic disorders, including metabolic syndrome, insulin resistance, hypertension, obesity, dyslipidemia, and type 2 diabetes [72]. Evidence indicates that dietary polyphenols can improve these conditions through multiple mechanisms. Compounds such as quercetin, resveratrol, and EGCG enhance glucose uptake via AMPK activation, improve pancreatic β-cell function, increase insulin secretion, and inhibit enzymes involved in carbohydrate metabolism, thereby improving insulin sensitivity. Aloe vera [127], grapes [128] and cinnamon [129], have shown beneficial effects on oxidative stress and glucose metabolism, while EGCG combined with caffeine may increase energy expenditure and fat oxidation [130]. Diets rich in polyphenols have also been associated with reduced obesity and metabolic syndrome risk, contributing to weight regulation and adipose tissue metabolism by transforming white adipose tissue into “brown” and enhancing energy consumption [131].

The liver is one of the main metabolic organs in the body, playing a central role in nutrient metabolism, detoxification, and energy homeostasis. Polyphenols also play an important role in liver function and metabolic detoxification, exhibiting hepatoprotective, antioxidant and anti-inflammatory effects. Several flavonoids and phenolic compounds—such as baicalin, curcumin, chlorogenic acid, resveratrol, silymarin, and ellagitannins—have demonstrated protective effects against toxin- or drug-induced liver injury by modulating pathways including ROS/MAPK, Nrf2 antioxidant responses, PI3K/AKT signalling and apoptosis regulation. Plant extracts from species such as *Microcos paniculata*, *Hibiscus sabdariffa*, *Phyllanthus amarus* and *Trigonella foenum-graecum* have also shown hepatoprotective effects in experimental models [132,133,134,135,136]. Clinical and cellular studies further suggest that flavonoids and other polyphenols can enhance liver detoxification processes by regulating oxidative stress, lipid metabolism, inflammation, and phase II detoxifying enzymes. Overall, these findings highlight the potential of polyphenol-rich diets to support metabolic health and liver detoxification, although further clinical trials are needed to determine optimal intake and long-term safety.

Recently, Subaş et al. reviewed the pathogenic mechanisms linking metabolic and gastrointestinal disorders, emphasizing the overactivation of the NLRP3 inflammasome, which triggers caspase-1 and promotes the release of proinflammatory cytokines (IL-1β and IL-18), and highlighted the therapeutic potential of dietary polyphenols as natural modulators of NLRP3 activity [137]. NLRP3 activation contributes to other gastric diseases such as Helicobacter pylori–related disorders by promoting IL-1β secretion and inflammatory cell infiltration, leading to chronic gastritis, ulcers and gastric cancer [138]. In liver diseases such as nonalcoholic fatty liver disease and alcohol-related liver disease, increased NLRP3, caspase-1, IL-1β, and IL-18 levels drive inflammation, fibrosis, cirrhosis, and hepatocellular carcinoma [139,140]. Elevated NLRP3 activity is also implicated in IBD, where it disrupts epithelial barriers, promotes intestinal inflammation, and increases colorectal cancer risk, although IL-18 may aid epithelial repair [141]. Additionally, NLRP3 activation underlies acute and severe pancreatitis by triggering caspase-1 and IL-1β–mediated pancreatic inflammation and tissue damage [142]. Collectively, these findings underscore the central role of NLRP3 in gastrointestinal pathologies and suggest that dietary polyphenols could offer a promising therapeutic strategy by modulating inflammasome activity.

### 6.3. Chronic Respiratory Diseases

Polyphenol supplementation has shown potential benefits in managing various respiratory conditions, including acute lung injury, pulmonary fibrosis, asthma, pulmonary hypertension, and lung cancer. In chronic obstructive pulmonary disease (COPD), compounds like baicalin, casticin, oroxylin A, fisetin, quercetin, resveratrol, and genistein exert anti-inflammatory and antioxidant effects, modulating NF-κB and Nrf2 pathways. In asthma, flavonoids such as apigenin, fisetin, and luteolin reduce Th2 cytokines (IL-4, IL-5, IL-13) and eosinophilia, decreasing bronchoconstriction and mucus production. For lung cancer, polyphenols including resveratrol, quercetin, EGCG, dieckol, kaempferol, and apigenin inhibit cell proliferation, oxidative stress, and EMT via PI3K/Akt, mTOR, and TGF-β/Smad3 signaling. In tuberculosis, EGCG and curcumin enhance pathogen clearance through apoptosis and autophagy. In ALI, polyphenols like curcumin, kaempferol, pinocembrin, and punicalagin reduce proinflammatory cytokines and oxidative stress, modulating NF-κB/MAPK pathways and supporting tissue repair [143]. Overall, polyphenol-rich diets or supplementation may offer therapeutic benefits across pulmonary diseases by regulating oxidative stress, inflammation, immune responses, and tissue remodeling, though further clinical studies are needed to define optimal dosing and long-term efficacy.

Excessive NLRP3 activation contributes to airway inflammation and tissue damage in chronic respiratory conditions, promoting immune cell infiltration, fibrosis, and airway dysfunction. In COPD, the inhibition of NLRP3, IL-1β, IL-18, or P2X7 receptor alleviates lung injury [144]. Curcumin and salidroside can improve the course of COPD by regulating blood pressure, inflammation, and the coagulation pathway. Mechanistically, polyphenol intervention can inhibit NLRP3 inflammasome activation, thereby reducing caspase-1–dependent IL-1β maturation and limiting inflammatory responses [145]. In bronchial asthma, NLRP3 activation by allergens, ATP, and ROS promotes IL-1β/IL-18 secretion, Th2 cell activation, and airway hyperresponsiveness, although some studies suggest context-dependent roles for IL-18 and NLRP3 in allergic responses [146]. Silicosis involves NLRP3-mediated macrophage activation, IL-1β secretion, and pulmonary fibrosis, with autophagy modulating inflammasome activity [147]. In bacterial infectious pneumonia, NLRP3 senses virulence factors such as α-hemolysin and pneumococcal hemolysin, triggering IL-1β and IL-18 release to coordinate host defense, with NLRP3 deficiency leading to impaired immune responses and increased mortality [148,149]. Collectively, these findings demonstrate that while NLRP3 activation can protect against infections, its excessive or chronic activation drives inflammation, tissue damage, and disease progression in the respiratory system.

### 6.4. Neurological and Neurodegenerative Diseases

Chronic neurodegenerative diseases such as Alzheimer’s disease, Parkinson’s disease, Huntington’s disease, amyotrophic lateral sclerosis and multiple sclerosis are strongly associated with oxidative stress, mitochondrial dysfunction, protein aggregation, and chronic neuroinflammation. Increasing evidence suggests that dietary polyphenols exert neuroprotective effects by modulating these pathological processes. Compounds such as resveratrol, curcumin, genistein, caffeic acid, fisetin, and kaempferol enhance autophagy and the clearance of toxic protein aggregates (e.g., amyloid-β, tau, α-synuclein, mutant huntingtin, and SOD1) through pathways including AMPK/SIRT1 activation and mTOR inhibition [150]. Together, these mechanisms highlight the potential of polyphenol-rich diets to help prevent or delay the progression of neurodegenerative disorders increasing brain plasticity and related cognition improvement. For example, polyphenols from lychee seeds, including rutin, quercetin, catechin, and proanthocyanidins, reduce Aβ-induced neuronal apoptosis and neuroinflammation by inhibiting NF-κB signaling and suppressing NLRP3 inflammasome activation. They also promote autophagy through the AMPK/mTOR/ULK1 pathway, decrease the expression of NLRP3-related proteins (NLRP3, ASC, caspase-1, IL-1β), and improve cognitive function in experimental Alzheimer’s disease models [151,152]. Overall, these findings suggest that polyphenols may help mitigate neurodegenerative progression by regulating NLRP3-mediated neuroinflammation and enhancing cellular protective mechanisms.

Across the disease contexts reviewed, a critical limitation that warrants explicit recognition is the heterogeneity of the experimental models employed and its implications for cross-disease comparisons and translational inference. Rodent models of atherosclerosis, predominantly the ApoE^−^/^−^ or LDLr^−^/^−^ mouse fed a high-fat Western diet, recapitulate lipid-driven vascular inflammation but do not adequately model the gut microbiota composition of human cardiovascular patients, as murine and human microbiota are substantially different in community structure and metabolic capacity. Models of metabolic dysfunction-associated steatotic liver disease, typically induced by high-fat or methionine-choline-deficient diets, reproduce hepatic steatosis and NLRP3-mediated fibrosis but compress disease timelines that in humans unfold over decades, potentially overestimating the anti-inflammatory potency of short-term polyphenol supplementation. Neurodegeneration models, such as the APP/PS1 transgenic mouse, reproduce amyloid pathology but do not capture the gut–brain axis dynamics characteristic of human neurodegeneration, where microbiota dysbiosis precedes and may drive central neuroinflammation over years. Models of IBD, including DSS-induced colitis, produce acute mucosal injury that diverges substantially from the chronic relapsing-remitting course of human Crohn’s disease or ulcerative colitis, in which NLRP3 plays context-dependent rather than uniformly pro-inflammatory roles. In respiratory models, instillation of silica particles or LPS produces acute innate immune responses that may not reflect the chronic low-grade NLRP3 activation characteristic of COPD or occupational lung disease. These distinctions do not invalidate the mechanistic findings reviewed, but they underscore the need for caution in extrapolating effect sizes and mechanistic conclusions across disease areas, and they highlight the urgency of model-specific human validation before clinical recommendations can be formulated.

## 7. Human Evidence Linking Polyphenol Intake to Inflammasome Modulation

While mechanistic studies in cell and animal models have provided detailed insight into the molecular pathways through which polyphenols modulate the NLRP3 inflammasome, a growing body of clinical and epidemiological evidence supports the translational relevance of these findings in humans. Several intervention trials and observational studies have documented reductions in circulating inflammatory biomarkers following polyphenol-rich dietary patterns or supplementation, with some data suggesting direct effects on inflammasome-related mediators.

The Mediterranean diet, rich in polyphenols from olive oil, fruits, vegetables, legumes, and red wine, has been most extensively studied in this regard. The landmark PREDIMED trial, a large randomized controlled trial conducted in Spain involving over 7000 participants at high cardiovascular risk, demonstrated that adherence to a Mediterranean diet supplemented with extra virgin olive oil or mixed nuts significantly reduced circulating levels of IL-6, CRP, and IL-18 compared to a low-fat control diet [153]. Notably, IL-18 is a direct downstream product of NLRP3-mediated caspase-1 activation, suggesting that the observed anti-inflammatory effects may partly reflect inflammasome suppression in vivo. Subsequent analyses from the PREDIMED cohort confirmed associations between high polyphenol intake and reduced concentrations of pro-inflammatory cytokines, including IL-1β [154]. Complementary observational data from the EPIC and Nurses’ Health Study cohorts have linked higher dietary polyphenol intake, particularly flavonoids and phenolic acids, with lower plasma CRP and IL-6 levels [155].

Resveratrol, a stilbene polyphenol found in grapes and red wine, has been evaluated in several human intervention trials. A randomized, double-blind, placebo-controlled trial in patients with type 2 diabetes showed that resveratrol supplementation (500 mg/day for 45 days) significantly reduced plasma IL-1β and IL-18 levels, accompanied by decreased NLRP3 mRNA expression in peripheral blood mononuclear cells [156]. Similar findings were reported in a clinical trial involving patients with metabolic syndrome, where resveratrol administration (150 mg/day for 12 weeks) significantly decreased circulating levels of high-sensitivity CRP, TNF-α, and IL-6, along with improved insulin sensitivity [157]. These results are consistent with the preclinical evidence implicating SIRT1 activation and NF-κB inhibition as central mechanisms by which resveratrol attenuates inflammasome priming.

Curcumin, the major bioactive polyphenol from turmeric, has also been studied in clinical settings. A meta-analysis of randomized controlled trials found that curcumin supplementation significantly reduced plasma CRP, IL-6, and TNF-α concentrations in patients with chronic inflammatory conditions including metabolic syndrome, non-alcoholic fatty liver disease, and rheumatoid arthritis [158]. A recent randomized trial specifically evaluated circulating IL-1β as a primary endpoint and reported a significant reduction following 12 weeks of curcumin supplementation in overweight individuals with elevated fasting glucose, consistent with NLRP3 inflammasome downregulation [159].

Quercetin, a flavonol abundant in onions, apples, and capers, has been shown in a randomized controlled trial to reduce plasma IL-6 and CRP in overweight subjects after 8 weeks of supplementation [160]. More directly relevant to inflammasome biology, a pilot clinical study in patients with gout, a paradigmatic NLRP3-driven disease, reported that quercetin supplementation attenuated IL-1β serum levels and improved clinical outcomes, supporting its potential as a dietary NLRP3 modulator [161]. Human studies with anthocyanins, particularly from berry extracts, have documented reductions in urinary and plasma inflammatory biomarkers including IL-6, IL-8, and oxidized LDL following 8–12 weeks of supplementation in healthy and at-risk populations [162].

Collectively, the human studies reviewed provide indirect but consistent evidence for inflammasome modulation by polyphenol-rich dietary patterns, primarily through reductions in the canonical NLRP3 outputs IL-1β and IL-18. However, several methodological limitations must be explicitly acknowledged when interpreting this body of evidence. First, none of the circulating biomarkers used as primary endpoints, including IL-1β, IL-6, CRP, TNF-α, and IL-18, is specific to NLRP3-mediated caspase-1 activation, as each can be produced through multiple inflammasome-independent inflammatory pathways; attributing observed reductions specifically to inflammasome suppression therefore requires caution. Second, several trials employed polyphenol supplements at doses substantially higher than those achievable through habitual dietary intake, limiting the generalisability of findings to food-based interventions and introducing a concentration gap analogous to that discussed for preclinical studies. Third, none of the trials reviewed stratified participants by gut microbiota composition or polyphenol metabotype at baseline; this represents an uncontrolled source of heterogeneity that likely accounts for a significant fraction of the variability in outcomes observed across studies. Fourth, follow-up periods ranging from 8 to 12 weeks in most trials may be insufficient to capture the full magnitude of microbiota-mediated effects, which evolve over longer timescales than direct pharmacological interventions. These limitations do not invalidate the clinical evidence reviewed, but they underscore the current inability to make definitive causal claims regarding NLRP3-specific modulation in humans and highlight the methodological upgrades required for future trials to provide mechanistically interpretable data (Table 4).

## 8. Discussion

The body of evidence reviewed across the preceding sections converges on a coherent mechanistic framework: the gut microbiota functions not merely as a passive recipient of dietary components, but as an active biochemical intermediary that determines the inflammatory consequences of diet at the systemic level. Within this framework, the NLRP3 inflammasome occupies a pivotal position, integrating microbial, metabolic, and redox-related signals into a unified inflammatory output. What emerges from the synthesis is not simply a catalogue of mechanistic interactions, but a conceptually unified model in which three interdependent layers, inflammasome biology, microbial ecology, and polyphenol biotransformation, operate as a single regulatory axis amenable to nutritional intervention.

The paradigm shift from a direct, antioxidant-centered model of polyphenol bioactivity to a microbiota-dependent, metabolite-driven one, detailed in Section 5, has profound implications that extend beyond mechanistic biology. If the primary effectors of polyphenol action are microbial catabolites rather than parent compounds, then the therapeutic value of a polyphenol-rich diet is inseparable from the metabolic capacity of the individual’s microbiota. This reframing fundamentally alters how clinical trials in this field should be designed and interpreted. The heterogeneous outcomes consistently observed across polyphenol intervention studies, where the same dietary intervention produces divergent effects in different individuals, are no longer simply attributable to compliance or bioavailability differences, but reflect the existence of distinct polyphenol metabotypes [93]. Individuals who do not harbor the bacterial taxa required for urolithin production will not generate this class of metabolites regardless of ellagitannin intake. This source of variability is currently uncontrolled in most clinical trials and represents one of the most significant methodological gaps in the field, underscoring the need for microbiota-stratified analyses as a standard component of future nutritional intervention studies.

A further conceptual contribution of this review is the identification of SCFA production as a mechanistically shared downstream pathway through which structurally diverse polyphenols converge on NLRP3 suppression. As described in Section “Polyphenols, Microbiota and Intestinal Barrier Integrity”, polyphenol-driven remodeling of the microbial community, specifically the enrichment of *Akkermansia muciniphila*, *Faecalibacterium prausnitzii*, and butyrate-producing *Firmicutes*, consistently enhances SCFA production irrespective of the specific polyphenol class consumed. The critical implication of this observation is that the overall dietary pattern, rather than any single polyphenolic compound, is likely the primary determinant of anti-inflammatory efficacy at the population level. This aligns with epidemiological data showing that composite dietary scores reflecting plant-food richness predict inflammatory biomarker reduction more robustly than intake of individual polyphenols, and it provides a mechanistic rationale for why Mediterranean-type dietary patterns outperform single-nutrient supplementation in large-scale cardiovascular trials.

The bidirectional relationship between gut dysbiosis and NLRP3 activity, in which dysbiosis drives inflammasome activation and activated inflammasome signaling in turn reshapes microbiota composition, generates a self-amplifying feed-forward loop [65]. From a therapeutic standpoint, this bidirectionality is significant: it implies that intervention at any node of the axis, whether through dietary polyphenols, prebiotic fibers, probiotic supplementation, or direct NLRP3 inhibition, has the potential to disrupt the loop and re-establish homeostasis. Nutritional modulation occupies a privileged position within this landscape because it simultaneously targets multiple nodes, microbiota composition, metabolite production, intestinal barrier integrity, and inflammasome priming, without the immunosuppressive risks associated with complete pharmacological NLRP3 blockade. A critical dimension of the evidence reviewed across all sections concerns the distinction between correlative and causal findings, which is insufficiently resolved at every level of the axis. The relationship between gut dysbiosis and NLRP3 activation is associative in humans and causal only within specific animal model systems; the methodological gap between species represents a fundamental translational barrier that germ-free and microbiota-transfer experiments, while invaluable, cannot fully bridge. The evidence linking polyphenol intake to reduced circulating inflammatory markers in human intervention trials is consistent but indirect with respect to NLRP3, relying on downstream cytokine proxies rather than direct inflammasome activity measurements. The claim that microbial metabolites are the primary bioactive effectors of polyphenol action, rather than parent compounds, is mechanistically compelling in experimental systems but has not been directly tested in controlled human studies through the administration of defined metabolites with and without concurrent microbiota modulation. Establishing causality in this field will require, at minimum, gnotobiotic validation of specific taxa and their metabolites combined with human studies measuring caspase-1 cleavage products, ASC speck formation, or gasdermin D processing as direct readouts of inflammasome activity, rather than the downstream cytokine concentrations that dominate the current clinical literature.

Nevertheless, important interpretive cautions are warranted. The majority of mechanistic evidence reviewed here derives from in vitro cell culture systems or preclinical rodent models, in which polyphenols and their metabolites are frequently administered at concentrations exceeding those achievable through physiological dietary intake. The translational relevance of these findings must therefore be assessed critically. While the mechanistic pathways identified are biologically plausible and internally consistent, their validation in human subjects using physiologically relevant exposure levels remains limited. Moreover, the NLRP3 inflammasome exhibits context-dependent roles: its activation is essential for host defense against infection and for tissue repair following injury, and its complete pharmacological suppression carries potential immunosuppressive risks [121]. Nutritional modulation, which produces more modest and physiologically graded effects compared to pharmacological inhibition, may therefore represent an advantageous strategy that rebalances rather than abolishes inflammasome activity.

A further consideration concerns the specificity of reported effects. Many polyphenols and their metabolites simultaneously modulate NF-κB, Nrf2, AMPK, and autophagy pathways that are not exclusive to NLRP3 regulation. Attribution of anti-inflammatory outcomes specifically to inflammasome modulation, as opposed to broader pleiotropic effects on innate immune signaling, requires careful experimental design including the use of NLRP3-deficient models, caspase-1 activity assays, and direct measurement of IL-1β and IL-18 maturation. Many of the studies reviewed here do not meet all of these criteria, and conclusions regarding NLRP3-specific effects should be interpreted with appropriate nuance.

An important mechanistic layer that emerges from this review is the role of SCFAs, particularly butyrate, as indirect mediators linking polyphenol-driven microbiota remodeling to NLRP3 inflammasome modulation. Although SCFAs are not direct biotransformation products of polyphenols, their production is consistently enhanced following polyphenol-rich dietary interventions as a consequence of the prebiotic-like restructuring of the microbial community, specifically the enrichment of *Akkermansia muciniphila*, *Faecalibacterium prausnitzii*, and other butyrate-producing taxa. SCFAs suppress NLRP3 activation through at least three converging mechanisms: epigenetic reprogramming via HDAC inhibition, GPCR-mediated anti-inflammatory signaling, and direct interference with inflammasome assembly through reduced mitochondrial ROS and potassium efflux [109]. The SCFA pathway is particularly relevant from a translational standpoint because it is shared across multiple polyphenol classes and food sources, suggesting that the overall dietary pattern, rather than any single polyphenolic compound, is the key determinant of anti-inflammatory efficacy. This observation aligns with epidemiological evidence showing that composite dietary scores reflecting overall plant-food richness are stronger predictors of inflammatory biomarker reduction than intake of individual polyphenols.

The mechanistic coherence of the microbiota–polyphenol–NLRP3 axis across organ systems is itself a significant finding: it suggests that this axis functions as a conserved regulatory module rather than a tissue-specific phenomenon. However, the degree of clinical validation varies substantially across disease areas. Evidence from human cohort studies and randomized controlled trials is most robust for cardiovascular and metabolic outcomes, where large-scale epidemiological data consistently associate polyphenol intake with reduced risk and where mechanistic biomarkers have been validated in interventional settings. In contrast, evidence for neurological and respiratory applications is predominantly preclinical, and the gut–lung and gut–brain axes, while mechanistically plausible and supported by compelling animal data, remain understudied in the context of polyphenol intervention in humans. These represent priority areas for future research, particularly as non-invasive biomarkers of neuroinflammation and pulmonary NLRP3 activity become increasingly accessible.

It is also important to note that for some conditions reviewed, particularly IgA nephropathy, AF, and HUA-associated renal injury, the evidence for gut microbiota–NLRP3 crosstalk is based on a limited number of studies, and the specific contribution of dietary polyphenols to these pathways has not been directly investigated. These disease contexts should be regarded as emerging areas of interest rather than established connections.

It is important to note that the current body of evidence is characterised by several methodological limitations that collectively constrain the strength of conclusions that can be drawn. Small sample sizes and short follow-up periods in clinical studies limit statistical power and the capacity to detect microbiota-mediated effects that unfold over timescales longer than typical pharmacological responses. The reliance of most mechanistic studies on transformed cell lines or primary cells from rodents may not faithfully reproduce the inflammatory signalling dynamics of human immune cells operating within a complex tissue microenvironment, particularly one continuously shaped by a living microbial community. The absence of standardised protocols for NLRP3 activity measurement across studies, with some using NLRP3 mRNA expression, others caspase-1 cleavage, and others cytokine secretion as readouts, prevents meaningful quantitative comparisons of effect sizes between polyphenol classes, metabolite types, and disease models, and introduces a risk of conflating mechanistically distinct phenomena under a common label. The predominant use of single-compound experimental designs does not reflect the complexity of dietary polyphenol exposure in vivo, where multiple structurally diverse compounds and their metabolites are simultaneously present and may interact synergistically or antagonistically in ways that cannot be predicted from single-compound experiments. Finally, the near-complete absence of longitudinal human data linking dietary polyphenol intake, microbiota composition, metabolite profiles, and NLRP3-related biomarkers in the same participants over time represents the most fundamental gap in the field: this is precisely the study design capable of establishing the temporal sequence of events required for causal inference, and its absence means that the axis, while mechanistically coherent, remains inferential rather than directly demonstrated in humans.

## 9. Conclusions

This review integrates evidence across molecular biology, microbiome science, nutritional biochemistry, and clinical medicine to support a unifying conceptual model: the gut microbiota–polyphenol–NLRP3 inflammasome axis represents a biologically plausible and increasingly supported regulatory network through which dietary patterns modulate innate immune tone, intestinal barrier integrity, and systemic inflammatory burden in chronic disease. However, it is important to emphasise that much of the current evidence derives from in vitro and animal studies, and direct validation in human populations remains limited.

Several key conclusions emerge from this synthesis. The biological activity of dietary polyphenols in the context of chronic inflammation appears to be mediated, at least in part, through their microbial catabolites rather than through direct systemic effects of parent compounds. The gut microbiota acts as a critical biochemical intermediary, generating urolithins, phenyl-γ-valerolactones, phenolic acids, and SCFAs, which have been shown in preclinical models to modulate NLRP3 signaling, mitochondrial function, and inflammatory gene expression.

The NLRP3 inflammasome is increasingly recognised as a mechanistic convergence point that integrates dysbiosis-derived priming signals with metabolic and redox danger cues, generating downstream inflammatory responses with consequences across multiple organ systems. While a bidirectional interaction between microbiota dysbiosis and NLRP3 activation has been proposed, much of the supporting evidence remains associative or derived from experimental models, and its relevance in humans requires further clarification. This interaction may contribute to the persistence of chronic inflammation and represents a potential target for dietary and microbiota-based interventions.

Interindividual variability in gut microbiota composition, reflected in the concept of polyphenol metabotypes, is likely to be an important determinant of the anti-inflammatory efficacy of polyphenol-rich diets. This variability has important implications for the interpretation of clinical trials and for the development of precision nutrition strategies. However, standardised definitions of metabotypes and their predictive value in human studies remain to be established. Future interventions should stratify participants by metabotype and integrate multi-omics profiling to improve the consistency and interpretability of outcomes.

**Figure 5:**
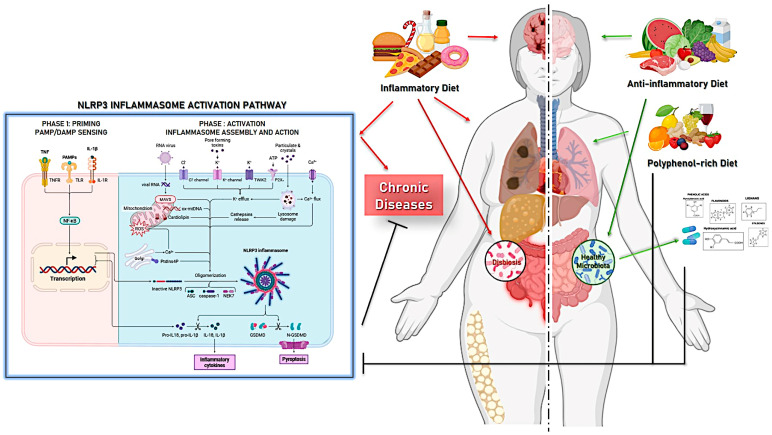
Systems-level regulatory network of the microbiota–polyphenol–NLRP3 axis. Dietary polyphenols shape gut microbial ecology and undergo microbial biotransformation into metabolites that regulate host pathways controlling NLRP3 inflammasome activity. In parallel, dysbiosis promotes inflammasome activation through endotoxemia, mitochondrial stress, and epithelial barrier dysfunction. Bidirectional feedback loops connect microbial ecology, inflammasome signaling, and chronic disease outcomes, supporting a network-based rather than linear model of regulation.

Collectively, the evidence reviewed supports the microbiota–polyphenol–NLRP3 axis as a genuinely integrated regulatory network, not a linear cascade: each of its three nodes, microbial ecology, polyphenol biotransformation, and inflammasome signalling, exerts reciprocal influence on the others, and intervention at any single node propagates effects across the system. This network character explains both the robustness of polyphenol-rich dietary patterns as anti-inflammatory strategies and the heterogeneity of clinical outcomes, which reflects individual-level variation in the microbial metabolic machinery. Translating this framework into clinical practice requires treating microbiota characterisation not as an exploratory variable but as a primary stratification criterion in nutrition trials, and measuring NLRP3-relevant biomarkers, IL-1β maturation, caspase-1 activity, circulating ASC specks, as mechanistic endpoints alongside traditional inflammatory markers. Only through this level of mechanistic resolution will the axis move from a conceptually coherent hypothesis to an actionable therapeutic target (Figure 5).

## 10. Future Perspective

A fundamental challenge limiting the translation of experimental findings into clinical practice is the marked interindividual variability in polyphenol biotransformation, which is largely determined by gut microbiota composition. The concept of polyphenol metabotypes has emerged as a critical framework for understanding why the same dietary exposure produces heterogeneous biological outcomes across individuals [101]. However, it remains unclear whether baseline metabotype classification can quantitatively predict the magnitude of NLRP3 inflammasome suppression following polyphenol-rich dietary interventions across diverse populations. A major bottleneck in this area is the lack of standardised and reproducible criteria for defining metabotypes across different omics platforms and cohorts. Future research must prioritise the systematic characterisation and validation of polyphenol metabotypes across diverse populations, integrating shotgun metagenomics, metabolomics, and host phenotyping. Longitudinal cohort studies are needed to determine whether metabotype classification at baseline predicts the anti-inflammatory efficacy of polyphenol-rich dietary interventions, and whether metabotype transitions induced by dietary or microbiome-targeted interventions translate into clinically meaningful changes in NLRP3-related biomarkers.

Beyond metabotyping, the ecological architecture of the gut microbiome itself warrants deeper investigation as a therapeutic target. The selective enrichment of keystone taxa such as *Akkermansia muciniphila*, *Lactobacillus* spp., and butyrate-producing *Firmicutes* through dietary polyphenols is well-documented, but the stability and durability of these shifts, and their relationship to inflammasome suppression, remain poorly characterised in humans. Critically, it is still unknown whether these microbial shifts play a causal role in NLRP3 modulation or simply represent correlates of broader dietary responsiveness. Addressing this question will require controlled human intervention studies combined with causal inference approaches and mechanistic validation in gnotobiotic models. Furthermore, the temporal stability of polyphenol-induced microbiota changes beyond the intervention period represents a key translational bottleneck. Synbiotic approaches combining targeted polyphenol supplementation with specific probiotic strains or prebiotic fibres represent a rational strategy to enhance microbial metabolite production and amplify anti-inflammatory outcomes, and deserve rigorous evaluation in controlled trials [163]. In particular, it remains to be established whether synbiotic interventions exert additive or synergistic effects compared with polyphenols alone, and whether their efficacy depends on baseline microbiome configuration.

The integration of multi-omics data into precision nutrition frameworks offers an unprecedented opportunity to move beyond one-size-fits-all dietary recommendations towards individualised interventions targeting the NLRP3 inflammasome axis. Large-scale dietary intervention trials powered for subgroup analyses based on microbiome composition, host genetics (including NLRP3 gene variants), and metabolomic signatures are urgently needed to identify responders and non-responders to polyphenol-rich diets. Artificial intelligence and machine learning tools applied to integrated multi-omics datasets hold promise for identifying predictive biomarker signatures and developing personalised dietary prescriptions with validated anti-inflammatory targets [164]. However, a critical unresolved issue is whether predictive models derived from multi-omics data in one population retain external validity across independent cohorts with different microbiome structures and dietary patterns. In addition, the lack of harmonised data preprocessing pipelines and standardised analytical frameworks represents a major barrier to reproducibility and clinical implementation.

A critical methodological priority is the development of validated, non-invasive biomarkers of NLRP3 inflammasome activity suitable for routine monitoring in clinical and population-based settings. Circulating IL-1β and IL-18, while informative, lack the specificity and sensitivity required for mechanistic inference. Emerging candidates include plasma caspase-1 activity, circulating ASC specks, gasdermin D cleavage products, and extracellular vesicle-associated NLRP3 components, all of which have been detected in peripheral blood and hold potential as surrogate endpoints in nutrition trials [165]. Nevertheless, it remains unclear which of these biomarkers most accurately reflects tissue-level inflammasome activation in vivo, and whether changes in circulating markers correlate with clinically meaningful outcomes. The absence of a validated gold-standard assay linking peripheral measurements to cellular inflammasome activity represents a major bottleneck for the field. Standardisation and validation of these biomarkers across cohorts and interventions will be essential for the field to advance towards evidence-based dietary guidelines that explicitly target inflammasome-mediated inflammation.

The convergence of mechanistic evidence, microbiome science, and early-phase clinical data positions specific polyphenols and their microbial metabolites as bona fide candidates for therapeutic development, particularly for conditions in which NLRP3 hyperactivation is a central pathological driver. Urolithin A, the ellagitannin-derived metabolite produced by gut bacteria, has advanced furthest along this translational trajectory: it has demonstrated efficacy in improving mitochondrial function and reducing inflammatory markers in randomised trials in healthy elderly individuals and in patients with metabolic disorders, and is already commercialised as a nutraceutical supplement [166]. However, it remains uncertain whether its efficacy is independent of host microbiome composition or whether interindividual variability in precursor metabolism still influences clinical response. This represents a key limitation for its generalisability as a therapeutic agent. Its mechanisms, mitophagy induction, NLRP3 inhibition, and Nrf2 activation, are directly aligned with the pathological processes underlying inflammasome-driven chronic disease, making it a prototype for microbiota-dependent polyphenol therapeutics.

A major obstacle to the therapeutic use of dietary polyphenols remains their inherently poor and variable bioavailability. Future research should explore advanced delivery systems, including nanoencapsulation, microbiome-sensitive formulations, and co-crystallisation with bioavailability enhancers, to ensure consistent and therapeutically relevant systemic exposure. A critical unresolved question is whether increasing systemic bioavailability translates into improved functional inhibition of the NLRP3 inflammasome, or whether local gut-derived metabolites remain the primary drivers of biological activity. An alternative and arguably more robust strategy is to bypass the bioavailability bottleneck altogether by targeting the microbiota to maximise endogenous production of bioactive polyphenol metabolites, rather than administering the parent compound. Notably, there is currently a lack of direct comparative studies evaluating formulation-based versus microbiome-targeted strategies, representing a key gap in the field. This microbiome-centric therapeutic paradigm, optimising the microbial metabolic machinery rather than the dietary molecule itself, aligns with the broader vision of precision microbiome medicine and may prove more reproducible across individuals with different baseline microbiota compositions [167].

Finally, the design of future clinical trials in this field must be substantially upgraded. Trials should incorporate mechanistic sub-studies measuring NLRP3-related endpoints in accessible biological matrices (blood, stool, urine), pre-stratify participants by microbiota composition and polyphenol metabotype, and employ adaptive or factorial designs to test synbiotic combinations. The integration of polyphenol-based nutritional interventions as adjuncts to existing pharmacological therapies for NLRP3-driven diseases, including gout, type 2 diabetes, cardiovascular disease, and IBD, represents a clinically realistic and immediately testable strategy that warrants exploration in well-powered multicentre trials [168].

## Figures and Tables

**Table 1:** Crosstalk between gut microbiota dysbiosis and NLRP3 inflammasome activation in multi-organ pathologies. ↓ Decrease in intracellular levels.

Disease	Gut Microbiota Alteration	NLRP3 Involvement	Pathophysiological Consequences
Neurodegenerative diseases	Dysbiosis, impaired intestinal barrier, increased permeability	Activation of NLRP3 in CNS via IL-1β and IL-18 maturation	BBB disruption, microglial activation, neuroinflammation, neurodegeneration
MASLD	Dysbiosis, increased gut permeability, translocation of PAMPs	NLRP3 activation in liver induces caspase-1–dependent IL-1β/IL-18 maturation	Hepatic inflammation, progression of liver injury
Acute Pancreatitis	Early dysbiosis; overgrowth of pro-inflammatory taxa (e.g., *Escherichia–Shigella*)	Intestinal NLRP3 activation exacerbates pancreatic injury	Increased neutrophil infiltration, necrosis, systemic inflammation
IBD	Altered microbial composition; mucosal imbalance	Dysregulated intestinal NLRP3 signaling	Barrier breakdown, excessive mucosal inflammation
Alcohol-induced neuroinflammation	Ethanol-induced dysbiosis	Hippocampal NLRP3 activation via systemic inflammatory signals	Microglial activation, depressive-like behavior
HUA-associated renal injury	Dysbiosis; increased uremic toxin production	Renal NLRP3 activation	Tubular injury, fibrosis, renal dysfunction
IgA Nephropathy	Reduced *Bifidobacterium*; microbial imbalance	Activation of NLRP3/ASC/caspase-1 pathway	Renal inflammation, proteinuria
AF	Age-associated dysbiosis; increased LPS	Cardiac NLRP3 activation	Atrial fibrosis, arrhythmogenic remodeling
AS	Dysbiosis; altered microbial metabolites	Vascular NLRP3 activation	Endothelial dysfunction, plaque progression
*S. aureus*-induced mastitis	Reduced secondary bile acids (↓ DCA)	DCA inhibits NF-κB and NLRP3 via TGR5-cAMP-PKA signaling	Increased inflammation and barrier damage when dysbiotic

**Table 2:** Microbiota-derived polyphenol metabolites and modulation of NLRP3 inflammasome signaling.

Microbial Metabolite	Primary Molecular Targets	Mechanisms of NLRP3 Modulation	Experimental Models
Urolithin A	Mitophagy pathways (PINK1/Parkin), mitochondrial ROS, TXNIP–NLRP3 interaction, p53–PGC-1α axis, caspase-1	Promotion of mitophagy; restoration of mitochondrial function; inhibition of caspase-1 activation; suppression of IL-1β secretion; reduction in pyroptosis	Microglia, macrophages, aging mice, neurodegeneration models
Phenyl-γ-valerolactones	NF-κB, MAPKs (ERK, JNK, p38), inflammatory cytokine signaling, cellular metabolism pathways	Inhibition of NF-κB and MAPK signaling; reduced transcriptional priming of inflammasome components; decreased inflammatory cytokine production; modulation of cellular metabolism	Macrophages, immune cell models
Protocatechuic acid	NF-κB, mitochondrial ROS, Nrf2 antioxidant pathway, caspase-1	Inhibition of NF-κB-dependent priming; reduction in mitochondrial oxidative stress; decreased NLRP3, ASC, and caspase-1 expression; reduced IL-1β release	Macrophages, endothelial cells, metabolic inflammation models
Anthocyanin-derived phenolic metabolites	Caspase-1, inflammasome sensors (NLRP3, NLRC4, AIM2), ROS signaling	Suppression of multiple inflammasome complexes; reduced caspase-1 activation; decreased IL-1β secretion; attenuation of neuroinflammation	Microglial cells

**Table 3:** Intestinal barrier–centered mechanisms linking microbiota remodeling, SCFAs, and NLRP3 inflammasome regulation.

SCFA	Primary Molecular Targets	Mechanisms of Action	Effects on Intestinal Barrier and Immunity	Consequences for NLRP3 Inflammasome
Butyrate	HDACs; GPR41 (FFAR3); GPR43 (FFAR2); GPR109A (HCAR2); NF-κB; mitochondrial ROS	Histone deacetylase inhibition; GPCR activation; modulation of cellular metabolism; antioxidant effects	Increased tight junction protein expression; improved epithelial energy metabolism; reduced intestinal permeability; anti-inflammatory cytokine production	Inhibition of NF-κB-dependent priming; reduced ROS-mediated activation; suppression of caspase-1 and IL-1β release
Butyrate (epigenetic immune regulation)	Foxp3 locus; HDACs	Epigenetic promotion of regulatory T cell differentiation	Expansion of Treg populations; immune tolerance; suppression of excessive inflammation	Indirect inhibition of inflammasome activation through immune regulation
Propionate	GPR41; GPR43; NF-κB; metabolic signaling pathways	GPCR-mediated immune modulation; regulation of inflammatory signaling; metabolic reprogramming	Enhancement of epithelial barrier function; modulation of immune cell responses	Reduced inflammasome priming signals through decreased inflammatory transcription
Acetate	GPR43; metabolic pathways; immune signaling mediators	GPCR activation; regulation of host metabolism; modulation of inflammatory responses	Support of epithelial homeostasis; modulation of immune cell activity	Indirect attenuation of inflammasome activation via reduced inflammatory tone
SCFAs (general metabolic effects)	Mitochondrial ROS; potassium efflux; ASC oligomerization	Regulation of mitochondrial function; reduction in oxidative stress; interference with inflammasome assembly signals	Reduced cellular stress signals and DAMPs	Direct or indirect suppression of NLRP3 activation

**Table 4:** Summary of human intervention and observational studies evaluating the effects of polyphenol-rich diets or supplementation on inflammatory and inflammasome-related biomarkers. The table reports study design, population, intervention, duration, and main outcomes, with particular emphasis on circulating cytokines (e.g., IL-1β, IL-6, IL-18, TNF-α) and related inflammatory markers. ↓ Decrease in intracellular levels.

Population	Intervention	Design	Duration	Main Outcomes
High CV risk	Mediterranean diet + EVOO/nuts	RCT	~5 years	↓ IL-6, CRP, IL-18
Type 2 diabetes	Resveratrol 500 mg/day	DB-RCT	45 days	↓ IL-1β, IL-18, ↓ NLRP3 mRNA
MetS patients	150 mg/day resveratrol	RCT	12 weeks	↓ IL-6, TNF-α, CRP
MetS, NAFLD, RA	Curcumin supplementation	Meta-analysis	variable	↓ CRP, IL-6, TNF-α
Overweight subjects	Quercetin supplementation	RCT	8 weeks	↓ IL-6, CRP
Gout patients	Quercetin	Pilot clinical study	short-term	↓ IL-1β
Healthy/at-risk	Berry extracts	RCT	8–12 weeks	↓ IL-6, IL-8, oxLDL

## Data Availability

The original contributions presented in this study are included in the article. Further inquiries can be directed to the corresponding author.

## Abbreviations

AF	Atrial fibrillation
AMPK	AMP-activated protein kinase
AS	Atherosclerosis
ASC	Adaptor protein apoptosis-associated speck-like protein
COPD	Chronic obstructive pulmonary disease
CVD	Cardiovascular disease
DAMPs	Danger-associated molecular patterns
DCA	Deoxycholic acid
EGCG	Epigallocatechin gallate
GPCR	G-protein–coupled receptors
HDAC	Histone deacetylases
HUA	Hyperuricaemia
IBD	Inflammatory bowel disease
IL-1β	Interleukin-1β
IL-18	Interleukin-18
LPS	Lipopolysaccharide
MAPKs	Mitogen-activated protein kinases
MASLD	Metabolic Dysfunction-Associated Steatotic Liver Disease
NF-κB	Nuclear factor kappa-light-chain-enhancer of activated B cells
Nrf2	Nuclear factor erythroid 2-related factor 2
NLRP3	NOD-like receptor family pyrin domain-containing 3
PAMPs	Pathogen-associated molecular patterns
PGC-1α	Peroxisome proliferator-activated receptor gamma coactivator-1α
ROS	Reactive oxygen species
SCFAs	Short-chain fatty acids
Treg	Regulatory T cell
UA	Urolithin A
